# Validation and Cultural Adaptation of the Parent Attitudes about Childhood Vaccines (PACV) Questionnaire in Arabic Language Widely Spoken in a Region with a High Prevalence of COVID-19 Vaccine Hesitancy

**DOI:** 10.3390/tropicalmed7090234

**Published:** 2022-09-08

**Authors:** Doaa Ali ElSayed, Etwal Bou Raad, Salma A. Bekhit, Malik Sallam, Nada M. Ibrahim, Sarah Soliman, Reham Abdullah, Shehata Farag, Ramy Mohamed Ghazy

**Affiliations:** 1 Family Health Department, High Institute of Public Health, Alexandria University, Alexandria 21561, Egypt; 2 School of Pharmacy, Lebanese International University, Beirut 961, Lebanon; 3 Environmental Health Department, High Institute of Public Health, Alexandria University, Alexandria 21561, Egypt; 4 Department of Pathology, Microbiology and Forensic Medicine, School of Medicine, The University of Jordan, Amman 11942, Jordan; 5 Department of Clinical Laboratories and Forensic Medicine, Jordan University Hospital, Amman 11942, Jordan; 6 Department of Translational Medicine, Faculty of Medicine, Lund University, 22184 Malmö, Sweden; 7 Nutrition Department, High Institute of Public Health, Alexandria University, Alexandria 21561, Egypt; 8 Tropical Health Department, High Institute of Public Health, Alexandria University, Alexandria 21561, Egypt; 9 Infectious Disease Residency Program, Egyptian Ministry of Health and Population, Cairo 71529, Egypt; 10 Biostatistics Department, High Institute of Public Health, Alexandria University, Alexandria 21561, Egypt; 11 Department of Family and Community Medicine, College of Medicine, King Khalid University, Abha 62529, Saudi Arabia

**Keywords:** immunization, parents’ beliefs, vaccine acceptance, SARS-CoV-2

## Abstract

The parents’ attitude toward vaccinating children and adolescents against coronavirus disease 2019 (COVID-19) remains inconsistent and needs further elucidation. The high rates of COVID-19 vaccine hesitancy in the Middle East and North Africa (MENA) region require intensive research to understand the determinants of this phenomenon. This study aimed to validate a version of the Parent Attitudes about Childhood Vaccines (PACV) tool in Arabic, the most widely spoken language in the MENA. The study objectives included the investigation of Arab-speaking parents’ views regarding COVID-19 vaccination of their children. Parents living in Egypt with at least one child aged 5–18 years were eligible to participate in the study that was conducted through an online survey with 15 PACV items. The PACV tool was translated into Arabic using forward and backward translation. To assess the psychometric properties of the Arabic version of PACV, Pearson’s correlation coefficient and exploratory and confirmatory factor analysis (EFA and CFA) were performed. A total of 223 parents participated in the study: 59.82% aged 30–39 years, 69.20% were females, 46.19% were university-educated, and 40.63% had one child. The overall Cronbach’s alpha for the Arabic version of PACV was 0.799. The EFA of the 15 items showed that three domains were most conceptually equivalent. All items had a positive significant correlation with the mean score of each subscale except for item 4 (r = 0.016, *p* = 0.811). Regression analyses results indicated that education, previous COVID-19 infection, vaccine status of parents, and PACV score were significantly associated with the intention of the parents to vaccinate their children against COVID-19. The CFA results showed that most of the factor loadings were statistically significant (*p* < 0.010) except for items 4 and 7. However, the root mean square error of approximation (RMSEA = 0.080) and the standardized root mean squared residual (SRMR = 0.080) indicated that the model had a reasonable fit, and the three factors were good in reproducing each correlation. Our study results indicated the validity and reliability of the PACV instrument in Arabic language. Consequently, the PACV can be used to assess COVID-19 vaccine hesitancy in a majority of MENA countries for better delineation of this highly prevalent phenomenon in the region.

## 1. Introduction

The devastating impact of coronavirus disease 2019 (COVID-19) pandemic has been manifested by the latest World Health Organization statistics, which reported more than 590 million confirmed cases with more than 6.4 million deaths as a result of the disease [1]. Large-scale vaccination against COVID-19 is regarded as the most promising approach to achieve population immunity, considering the currently limited effective medication options and the incessantly increasing economic burden of the pandemic [2,3].

With the emergence of new severe acute respiratory syndrome coronavirus 2 (SARS-CoV-2) variants with high transmissibility, such as the Omicron variant, many countries expedited vaccination with booster doses and extended immunization campaigns towards children and adolescents [4]. Individuals younger than 20 years of age who have been infected with SARS-CoV-2 represented up to 21% of national COVID-19 caseloads across 103 countries as of March 2022 [5].

Vaccination against SARS-CoV-2 infection has been debatable for adolescents aged 12–15 years and even more controversial for children under 12 years of age [6]. Despite the large number of cases among children and adolescents in some countries, COVID-19 generally poses a minor risk to this age group, with less than 2% of symptomatic cases requiring hospitalization [7,8,9]. As for mortality figures, age-disaggregated data reported to the WHO as of October 2021 showed that children and young adolescents aged 5–14 years accounted for 0.1% of the total global deaths from COVID-19 [10]. However, there are several arguments in favor of vaccinating children and adolescents. For example, vaccination can protect against prolonged COVID-19 symptoms, referred to as “long COVID-19”, which can develop even after mild or moderate SARS-CoV-2 infections [11,12,13]. It can also guard against pediatric inflammatory, multisystem syndrome temporarily associated with SARS-CoV-2 (PIMS-TS), a hyperinflammatory condition that can complicate recovery from COVID-19 [14,15]. Vaccinating children can further help to avoid the closure of educational facilities, which is beneficial since lockdowns were shown to detrimentally impact children’s physical and mental health [16]. Possible advantages of COVID-19 vaccination in children extend to involve the establishment of population immunity with reduction in virus circulation and lower possibility of emergence of virus variants [17]. Nevertheless, disadvantages of COVID-19 vaccination in children should be considered, including: limited vaccine supplies in some countries with importance of prioritizing high-risk groups; possible adverse events following vaccination in children; and lower incidence of infection, severe disease, and mortality among children [17].

Several widely used COVID-19 vaccines have been granted emergency use authorization or full approval for children under 18 years of age in at least one country [18]. These include the U.S.-based Pfizer-BioNTech COVID-19 vaccine for children aged 5 years or older [19], Moderna (Spikevax) COVID-19 vaccine for children aged 12 years or older [20], China-based Sinopharm BBIBP and Sinovac-CoronaVac COVID-19 vaccines for children as young as 3 years [21], and India-based Covaxin for children aged 12–18 years [21].

Immunizing children and adolescents largely depend on their parents’ or guardians’ decision. Findings of meta-analyses suggest that parents’ willingness to have their children receive a COVID-19 vaccine (61%) is lower than the general population’s intention to get vaccinated (73–75%) [22,23,24]. Higher rates of COVID-19 vaccine hesitancy have been associated with the following variables: younger age, low income, low educational level, high reliance on social media as a source of information regarding the vaccines, low perceived risk from COVID-19 (high levels of complacency), low trust in scientists (low levels of confidence), and belief in conspiracy theories [24,25,26]. The investigated factors that were linked to parents’ decision not to vaccinate their children against COVID-19 fall along the same lines [22].

The current and previous evidence points to the high prevalence of COVID-19 vaccine hesitancy in a majority of Arab countries in the Middle East and North Africa (MENA) region [27,28]. For example, a study conducted across 13 Arab countries showed that only 27% of the respondents were confident regarding COVID-19 vaccines [29]. Nevertheless, there is a shortage of studies evaluating parental attitudes towards COVID-19 vaccination in children in the Arab-speaking countries, which suggests a further need for such studies in the region. A strong predictor of parents’ acceptance to vaccinate their child against COVID-19 is their intention to receive the vaccine themselves [22,30]. The current COVID-19 immunization figures in the MENA region are relatively low, with <50% of the population having received full vaccination [31]. Therefore, this observation is expected to be reflected upon the figures relating to acceptance of COVID-19 children vaccination in the region.

In Egypt, about 36% of the adult population completed their initial vaccination, while 12% are only partially vaccinated [32]. The Egyptian national vaccination program began in January 2021, and the following vaccines were used: Pfizer-BioNTech, Oxford–AstraZeneca, Moderna, Johnson & Johnson’s Janssen COVID-19 Vaccine, Sputnik V, Sinopharm, and Sinovac, with the request from the public to register on an Egyptian government website to get vaccinated (www.egcovac.mohp.gov.eg, accessed on 7 September 2022). Egypt approved Pfizer-BioNTech COVID-19 vaccine for children aged 12 to 15 on 28 November 2021.

The Parent Attitudes about Childhood Vaccines (PACV) is a valid tool that has been successfully used in many countries to delineate the parental vaccine hesitancy [33,34,35,36,37]. It is a useful tool to predict under-immunization, particularly for children with parents having high PACV scores. Our study aimed to develop a validated Arabic version of the PACV survey instrument to collect COVID-19-related vaccination data in the Arab world. Since COVID-19 vaccination has been authorized for children aged 12 years and above in many Arabic countries [38,39], we aimed to use the survey to determine the extent of parents’ hesitancy towards vaccinating their children against COVID-19 in the Arab region.

## 2. Materials and Methods

### 2.1. Study Design

A predesigned self-administered questionnaire was developed in addition to the PACV scale using Google Forms to collect the data. Participants were invited to participate through different social media platforms including Facebook, WhatsApp, and Twitter (Supplementary S1).

Parents living in Egypt who had at least one child aged 5–18 years were eligible to participate in the study. Based on the sample size recommendations of having 10 participants respond to each item for validating a questionnaire (ratio of 10–15:1), we collected a total of 223 responses to assess the psychometric properties of the PACV tool in Arabic [40].

The study was approved by the Ethics Committee of the Faculty of Medicine, Alexandria University, Egypt, following the International Ethical Guidelines for Epidemiological studies.

### 2.2. Validation of the PACV Questionnaire in Arabic Language

#### 2.2.1. Forward and Backward Translation

The original version of PACV is not under copyright restriction. The guidelines for translation and cross-cultural adaptation were followed [41]. The tool was forward translated into Arabic by two bilingual translators whose mother tongue was Arabic. The translators were health professionals who were aware of the concepts examined by the questionnaire. After revisions, two bilingual translators whose mother tongue was English translated the tool backward into English (Supplementary S2) [42]. Discrepancies between the original source and the back-translated version were discussed. The bilingual expert panel altered the forward translated tool as many times as needed until a satisfactory version was reached. Standard Arabic was the language used in this study since it is the official language of 21 Arab countries in the MENA region and based on the fact that standard Arabic is widely taught, understood, and spoken by the native Arabs [43].

#### 2.2.2. Content Validity and Expert Evaluation

Content validity refers to the extent to which the items in a questionnaire are representative of the entire theoretical construct the questionnaire is designed to assess [44]. Content validity was performed over several steps in our study. First, the content validation form was prepared to ensure that the review panel had a clear understanding of the task. Second, the review group responsible for reviewing the questionnaire was selected based on the reviewers’ expertise in vaccination. The committee consisted of 4 reviewers: D.A.E. (Family Health), S.A.B. (Public Health), R.M.G. (Pediatrician), and S.S. (Tropical Health).

#### 2.2.3. Pilot Testing and Cognitive Interviewing

After translating the questionnaire, trained members of the research team (S.S. and R.A.) conducted cognitive interviews among 16 participants of the intended respondents to evaluate readability, language, wording, cultural appropriateness of the items, clarity of the instructions for each section, as well as the ease of participants’ understanding of the questions. The research team reformulated the Arabic questions (Supplementary S3). Finally, the translated version was approved by the researchers and was ready for field testing. The final survey form is presented in (Supplementary S4).

#### 2.2.4. Score Interpretation, Data Management, and Psychometric Analysis

The PACV tool consists of 15 items; we considered the score < 21 as “non-hesitant” and that ≥21 as “hesitant” [45].

Quantitative variables were summarized as mean ± standard deviation (SD), while qualitative variables were presented with percent and frequency. Mean scores for each subscale were calculated. Cronbach’s alphas were calculated for the sub-scales of the questionnaire to assess their internal consistency [46]. Simple logistic regression was computed to estimate the unadjusted odds ratio (OR) with a 95% confident interval (CI) and to estimate the effect of each individual predictor, including the dichotomized PACV scale (hesitant/not hesitant) on parents’ intentions to vaccinate their children against COVID-19.

Construct validity is defined as the “extent to which an instrument assesses a construct of concern and is associated with evidence that measures other constructs in that domain and measures specific real-world criteria” [40]. It is determined using content, criterion-related validity, and structural or factorial validity. Concurrent, convergent, and divergent validity were used as indicators of criterion-related validity. Concurrent validity was assessed by determining whether the PACV scale predicted the intention to vaccinate children against COVID-19 through multiple logistic regression analysis. We included in the model ‘‘the intention to give the COVID-19 vaccine” as the dependent variable and the dichotomized PACV score with the baseline criteria of the study participants as independent variables.

Convergent validity was assessed by analyzing inter-item and item-to-mean scores of the sub-scale correlation. Discriminant (divergent) validity was evaluated by calculating the factor correlation matrix of the three subscales. Pearson’s correlation analysis was used to calculate the inter-item and item-to-mean score of the sub-scale correlation. The exploratory factor analysis (EFA) aimed to identify the major factor structures for the set of 15 items and determine the number of latent factors without making assumptions about the factor relationships [45]. Kaiser–Meyer–Olkin (KMO) sampling adequacy measure and Bartlett’s sphericity test were performed before conducting EFA [47]. The decision pertaining to item factor loading was based on the scree plot, eigenvalues, percentage of variance in the items, and repeat component matrix analysis [45]. We ran EFA using the principal component analysis (PCA) with Promax rotation to calculate the inter-factor correlation. Discriminant validity was assessed if inter-factors correlation based on the factor correlation matrix were less than 0.7. A factor loading cut-off value of 0.50 was chosen to decide which items were highly associated with a given factor. In interpreting the output, we opted to use this criterion: each factor should have at least 2 items with high factor loadings of 0.5 and higher on the primary factor and minimal cross-loadings on any of the other factors (a < 0.2) to reduce the overlap between the sub-scales.

### 2.3. Statistical and Confirmatory Factor Analysis

We used the statistical software for data science (STATA) and the statistical package for the social sciences (SPSS) AMOS 26 to run the analyses. The *p*-value < 0.050 was considered statistically significant.

The confirmatory factor analysis (CFA) that was performed based on the selected participants aimed to measure how well the factor structure identified in the EFA fits the observed data. Specifically, we assessed the convergent and discriminant validity of the constructs and model fit measures using the ¨structural equation modeling (SEM) technique.

## 3. Results

### 3.1. Characteristics of the Study Participants

The baseline characteristics of the study population are shown in (Table 1). Most participants were females (69.20%), 30–39 years of age (59.82%), university-educated (46.19%), and had one child (40.63%). More than half the participants worked in governmental sectors (66.96%), had enough income (60.27%), had an extended family (28.7%), had a family size of ≥5 (49.11%), and were health-insured (79.02%). Most of the participants (75.90%) were healthy with no history of chronic disease, had previously contracted COVID-19 (42.86%), and reported receiving the first and second doses of COVID-19 vaccine (43.64%). Notably, more than half the study participants (56.25%) did not favor administering the COVID-19 vaccine to their children. According to parents’ reports, most children and adolescents had no chronic diseases (94.20%), contracted COVID-19 (67.41%), and had received their scheduled vaccines (72.77%) except for the flu vaccine, with 75.00% who did not receive this vaccine. Interestingly, 92.44% of the parents scored ≥ 21 on the PACV questionnaire and were thus identified as “hesitant” to have their children receive the COVID-19 vaccine.

### 3.2. Predictors of Parental COVID-19 Vaccination Hesitancy

There were significant associations between being hesitant to vaccinate children against COVID-19 and being a female (OR = 1.94, CI = 1.09–3.44, *p* = 0.020), being undergraduate (OR = 5.45, CI = 2.07–14.33, *p* = 0.001), being unemployed (OR = 2.84, CI = 1.20–6.73, *p* = 0.017), and having no previous or no documented COVID-19 infection (OR = 0.36, CI = 0.19–0.68, *p* = 0.020) (OR = 0.46, CI = 0.23–0.88, *p* = 0.020), respectively. Similarly, taking the first COVID-19 vaccine dose and waiting for the second (OR = 0.13, CI = 0.03–0.58, *p* = 0.007), taking two doses and awaiting the booster dose (OR = 0.12, CI = 0.08–0.37, *p* < 0.001), and taking all three doses (OR = 0.08, CI = 0.02–0.30, *p* < 0.001) were significantly associated with the intention to vaccinate children against COVID-19. Finally, the total PACV score (OR = 11.20, CI = 2.50–50.28, *p* = 0.002) was another factor positively associated with the intention to have children vaccinated (Table 2).

A binary logistic regression model showed that the main predictors for vaccine acceptance were being educated (OR = 3.58, CI = 1.02–11.70, *p* = 0.045), having undergone postgraduate studies (OR = 1.57, CI = 0.40–6.05, *p* = 0.051), having not previously contracted COVID-19 (OR = 0.25, CI = 0.10–0.58, *p* = 0.002), being unsure about having previously contracted COVID-19 (CI = 0.11–0.62, *p* = 0.002), taking the first dose and waiting for the second dose (OR = 0.07, CI = 0.01–0.46, *p* = 0.005), taking the first and the second doses of the vaccine and awaiting for the third dose (OR = 0.08, CI = 0.02–0.35, *p* = 0.001), and taking the three doses of the vaccine (OR = 0.04, CI = 0.01–0.23, *p* < 0.001). Interestingly, parents who were hesitant using the PACV scale had around 11 times the odds of having no intention to vaccinate their children against COVID-19 than unhesitant parents (OR = 10.8, CI = 1.92–40.6, *p* = 0.007, Table 3).

The mean PACV score was 26.68 ± 4.46 (range: 16.00–42.00). The overall Cronbach’s alpha for parents’ hesitancy towards COVID-19 vaccination using PACV was 0.80. The Cronbach’s alpha for each of the domains “Attitude”, “Safety and Efficacy”, and “Behavior” were 0.74, 0.82, and 0.57, respectively. The mean score for all questions that showed positive and significant correlation with the mean score of each subscale indicated that the questionnaire had good convergent validity except for question 6 (r = 0.016, *p* = 0.811, Table 4).

There were no correlation coefficients larger than 0.7; hence, the factors derived from EFA revealed adequate discriminant validity (Table 5).

### 3.3. Factorial Analysis

**Figure 1:**
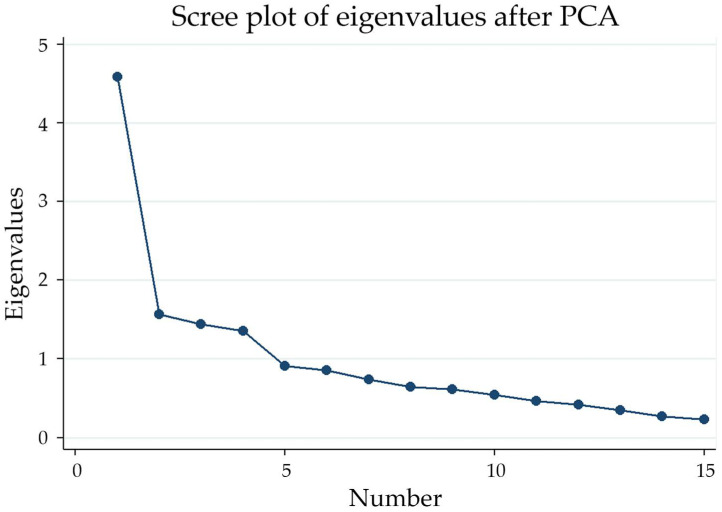
Scree plot of the Arabic version of the Parent Attitudes About Childhood Vaccines (PACV) scale. PCA, principal component analysis.

Eigenvalues showed that four factors were >1, with a total variance of 60%. However, based on the scree plot, the elbow of the curve occurred at the second endpoint, and the drop from the first, the second point to the second endpoint was more substantial than other data points (Figure 1). Therefore, a repeat component matrix analysis was utilized with factors fixed at two and four, and the three-factor solution was deemed to be most conceptually appropriate.

**Figure 2:**
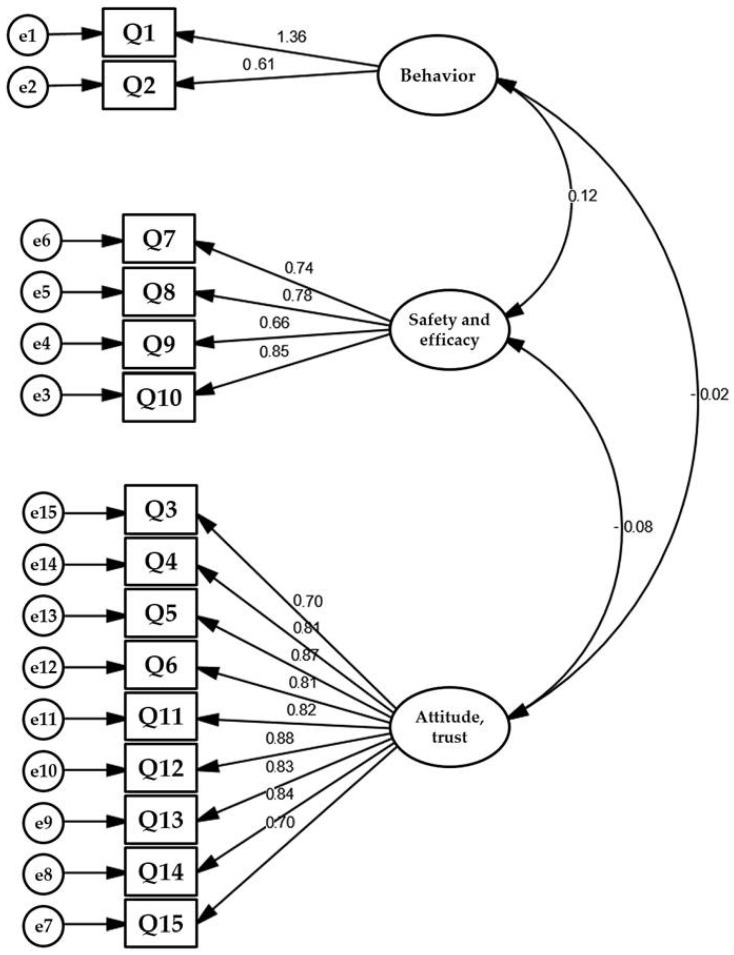
Confirmatory factor analysis of the 15 questions related to the three domains of the Arabic version of the Parent Attitudes About Childhood Vaccines (PACV) scale.

In Table 6, presentation of the factor loadings, subscale, and labeling for the PACV Arabic version is provided. The EFA with Promax rotation showed that items 3, 5, and 11–15 have very good convergent (>0.5) on factor 1 “Attitude” and discriminate validity (<0.2) on other factors. Items 8 and 9 also showed very good convergent and discriminate validity on factor 2, “Safety and efficacy”. Item 10 has acceptable convergent and discriminate validity on “Safety and efficacy”. Items 1 and 2 also showed very good convergent and discriminate validity on factor 3 “Behavior”. As for items 4, 6, and 7, they showed low convergent and discriminate validity and high uniqueness (low communality, Figure 2).

All the loadings ranged between 0.66 and 0.94. The construct reliability of the five factors in the CFA final model were above the range of 0.70 to 1.36. For convergent validity, the average variance-extracted (AVE) values of confidence, complacency, and calculations factors were above 0.50. Although the AVE value of constraints and collective responsibility factors were less than 0.50, the factors’ specific items loadings were acceptable for convergent validity since there were no items with loading below 0.40. The correlation between the five latent variables was less than the squared root of AVE; hence, this could not be problematic with discriminant validity. An overview of goodness-of-fit measures for the final model is presented in Table 6. The results demonstrate good model data fit, i.e., RMSEA 0.9 and SRMR.

## 4. Discussion

To the best of our knowledge, an Arabic-validated instrument that can evaluate parental hesitancy towards COVID-19 vaccination in the Arab world does not exist. In this paper, we validated the PACV questionnaire in Arabic. The differences between populations and cultures necessitate the assessment of the reliability and validity of survey instruments [41].

Arab populations have different dialects; however, standard Arabic is the official written language regardless of the geographical location. Therefore, we used standard Arabic to translate and validate the PACV questionnaire among Egyptians. This Arabic tool is the first to undergo a thorough cross-cultural adaptation, translation, and validation process based on recommended guidelines [42]. In the United Arab Emirates (UAE), Al Suwaidi et al. developed an Arabic version of the PACV tool; however, they only conducted forward and backward translations and calculated the Cronbach’s alpha for the Arabic PACV scores [48].

During the forward and backward validation process, most questions were clear and easy to understand except for the word “shot”, which we replaced with the words “vaccination doses”. In addition, in question 6, we replaced “to get a shot” with “rather than getting vaccinated” to avoid any confusion. Finally, in question 7, the phrase “to get fewer vaccines” was not fully understood, so we added the word “doses” to alter the phrase into “to get fewer doses of vaccines”. To check for translation quality and the practical aspects of test administration, the translated scale was then pilot-tested with an Arabic-speaking individual, who deemed it functional and the information suitable. We faced challenges in translating sentences such as “children get more shots than are good for them” and “it is better for children to get fewer vaccines at the same time”, as they had no typical Arabic equivalent. Some participants commented on the question, “I am able to openly discuss my concerns about shots with my child’s doctor” with the information that they were not following up with a pediatrician anymore. 

In this study, the Arabic version of PACV—made up of 15 questions—was organized into three factor domains: “Attitudes”, “Safety and efficacy”, and “Behavior”. This is identical to the original questionnaire, which had 15 items divided into three categories [34]. The psychometric results of the Arabic version of the PACV were close to the values of the corresponding items in the Malay-validated version [49]. Overall, the value of Cronbach’s alpha was 0.799, which meant it was stable and reliable over time. A low value for Cronbach’s alpha was obtained from the behavior sub-scale (0.573), which was still acceptable; however, the Arabic version of the questionnaire showed a high Cronbach’s alpha (0.74) for the attitude sub-scale and safety and efficacy (0.82). Similarly, in the Malay version, the value of Cronbach’s alpha was 0.77 and 0.54, 0.77, and 0.81 for each domain separately. The relatively low value of the behavior domain may be explained by the different context in which we tested the PACV tool. Furthermore, while the original PACV was tested before the era of the COVID-19 pandemic, our questionnaire was peculiarly validated for COVID-19 vaccination purposes. The debate about the different vaccines’ efficacy and safety have influenced the Arab population’s acceptance. In addition, the vaccines are not widely administered to all children in all countries due to different policies regarding the eligibility and stock availability.

In this study, the number of latent constructs (3) discovered through factor analysis that corresponded to these sub-domains was comparable to the number of PACV content domains identified a priori [48]. However, during content validation, two items from the “Behavior” subdomain were identified as items with formative scale and excluded from EFA but retained as part of the demography. Three items were deleted due to poor factor loading of <0.3. Therefore, the validated final PACV-Malay version consisted of 12 items framed within three factor domains (a novel item was added) [49]. Another large study conducted in three languages (Italian, French, and German) to identify the subdomains of the PACV using CFA and Moken scale analysis found that the German tool had 13 items, the French had 14 items, and the Italian tool had 11 items loaded on a single factor [47].

Our research also revealed intriguing findings concerning parental views about childhood vaccination. We discovered that a higher number of parent respondents (92.4%) had denied or delayed the recommended COVID-19 vaccines. In the same way, Chen et al. conducted a meta-analysis on 29 studies (N = 68,327 people) chosen from 452 identified records [22]. The estimated global vaccine acceptance rate was 61.4% (95% CI: 53.56–68.69%, I2 = 99.3%), with countries and regions ranging from 21.0% to 91.4 % [22]. Our analysis showed that parents who contracted COVID-19 tended not to vaccinate their children against COVID-19, and this may be justified by the perception of severity for people who became infected and had no symptoms and thus felt there was no need to give the COVID-19 vaccine to their children. Interestingly our findings also showed that parents with university education tended not to give the vaccine to their children, and this was also related to the perception of severity and efficacy. Our findings further support the notion that an increasing overall score on the 15-item PACV is related to increased under-immunization. As a result, the improved PACV appears to accurately assess the underlying construct of vaccination reluctance.

Studies on parental attitude towards COVID-19 vaccination were conducted recently in the Arab countries of the MENA region. A noteworthy finding by Khatatbeh et al. was that according to the parent-reported coverage of COVID-19 vaccination in children, 32% vaccinated their children against COVID-19 [50]. This result reported in eight MENA countries (Iraq, Jordan, Kuwait, Lebanon, Palestine, Qatar, Saudi Arabia, and the United Arab Emirates (UAE)) was much higher than the estimated proportion of non-hesitant parents reported in this study (8%). Likewise, Almalki et al. investigated parental COVID-19 vaccine hesitancy using the health belief model [51]. The study that was conducted in Saudi Arabia reported parental vaccine hesitancy for children aged 5 to 11 years at a rate of 62%, which was lower compared to the findings of the current study (92%). Low confidence in vaccine safety or efficacy were the most relevant factors to be associated with parental COVID-19 vaccine hesitancy in the Saudi study [51]. On the other hand, a lower rate of parental vaccine hesitancy was reported by Al Suwaidi et al. in the UAE, with only 12% of parents classified in the hesitant group [48].

The literature addressing the determinants of COVID-19 vaccine hesitancy developed at a swift rate, which helped to understand the possible determinants of this concerning phenomenon [27,52,53,54,55,56]. Indeed, promoting vaccination against COVID-19 necessitates understanding whether people are willing to be vaccinated, the factors associated with their attitude toward COVID-19 vaccination, and the most trusted sources of information in their decision making [52]. Interestingly, our findings showed that parents with university education and postgraduates tended not to give the vaccine to their children. This finding is consistent with a previous study, which found that undergraduate parents are more enthusiastic about vaccinating their children than higher-educated parents [50]. Conflicting results were reported regarding the role of educational level in parental willingness to vaccinate their children against COVID-19, which mandates future studies to understand the role of education in parental attitude towards COVID-19 vaccination [57,58,59].

In this study, neither age nor sex of the parents were significant determinants of COVID-19 vaccine hesitancy. To the contrary, children’s vaccination was found to be significantly related to the age of the parents in the recent study by Khatatbeh et al., with older participants showing lower levels of vaccine hesitancy [50]. On the other hand, the study by Almalki et al. showed that females were more hesitant to vaccinate their children against COVID-19 [51]. Therefore, a better depiction of the role of age and sex of the parents should be considered in any future work addressing parental vaccine hesitancy.

### Strengths and Limitations

Our study’s strength is that it is the first to validate the PACV questionnaire for utilization in assessing COVID-19 vaccination hesitancy. In addition, we performed confirmatory factor analysis that confirmed the loading of different variable on the domain. Other similar studies that validated the PACV did not conduct CFA, even including the original study that developed this tool.

We recognize, however, that there are a few limitations that should be considered as follows: First, the study was carried out in the form of a web-based survey, which may have led to selection or non-response bias. It was, nevertheless, in line with the study objectives since it supervised the large-scale survey administration during a time when restrictions were in place. This strategy protected both interviewers and interviewees. Because of the prolonged lockdown and limited access to community members, this was the best option. Second, because the study was cross-sectional, it did not allow for an assessment of changes in COVID-19 vaccine acceptability over time, following broad efforts to persuade people to obtain the vaccination. However, we assumed that it would have no effect on the stability of replies because the Arabic version of PACV showed good dependability. Third, we did not assess the validity of the PACV questionnaire among Arabs residing in other Arab nations; nonetheless, as previously stated, formal Arabic is the most extensively spoken language in the area. Finally, we employed a non-random sampling strategy (convenient sampling method) to include the research population; nonetheless, due to the limited access to community members, this method was the most suitable.

Future work assessing vaccine hesitancy in general and parental vaccine hesitancy in particular is recommended to assess the religious, spiritual, and ethical aspects involved. This comes in light of previous evidence of their discernible role as determinants of vaccination hesitancy [60,61].

## 5. Conclusions

The validated Arabic version of the PACV has good reliability and validity to be used to assess the parent attitude toward vaccination. The validity of this tool can pave the way for large-scale studies in the Arab-speaking countries of the MENA region, where COVID-19 vaccine hesitancy is highly prevalent.

## Figures and Tables

**Table 1:** Baseline characteristics of the study population and the Parent Attitudes About Childhood Vaccines scores.

Variables	Category	N (%)
Age in years	18–29	15 (6.70)
	30–39	134 (59.82)
	40–49	62 (37.68)
	≥50	13 (5.80)
Sex	Male	69 (30.80)
	Female	155 (69.20)
Education	Below university education	25 (11.21)
	University education	103 (46.19)
	Postgraduate	95 (42.60)
Number of children	One child	91 (40.63)
	Two children	83 (37.05)
	Three children	39 (17.41)
	Four children	11 (4.91)
Relation to the child	Mother	155 (69.20)
	Father	69 (30.80)
Place of work	Government	150 (66.96)
	Private	42 (18.75)
	Not employed	32 (14.29)
Health-insured	Yes	177 (79.02)
	No	47 (20.98)
Income	Not enough; on a loan and cannot pay back	15 (6.70)
	Not enough; on a loan but can pay back	48 (21.43)
	Enough	135 (60.27)
	Enough and saving	26 (11.61)
Older adults living in the same home	Yes	64 (28.70)
	No	159 (71.30)
Family size	2	12 (5.36)
	3–4	102 (45.54)
	≥5	110 (49.11)
Previous COVID-19 infection	Yes	96 (42.86)
	No	68 (30.36)
	Not sure	60 (26.79)
COVID-19 vaccine status	Does not want to take the vaccine	30 (13.64)
	Took the first dose and is awaiting the second	15 (6.82)
	Took the first dose but does not want to take the second dose	3 (1.36)
	Took the first and second doses and is awaiting the booster dose	96 (43.64)
	Took the first and second doses but did not want to take the booster dose	31 (14.09)
	Took the three doses	31 (14.09)
	Wants to take the vaccine, but it is not scheduled yet	14 (6.36)
Parent with chronic diseases	Yes	54 (24.11)
	No	170 (75.89)
Children with chronic disease	Yes	13 (5.80)
	No	211 (94.20)
Children received scheduled vaccines	Yes	163 (72.77)
	No	61 (27.23)
Children received influenza vaccine	Yes	51 (22.77)
	No	168 (75.0)
	I do not know	5 (2.23)
Children with previous COVID-19 Infection	Yes	31 (13.84)
	No	151 (67.41)
	I do not know	42 (18.75)
Parents intentions to allow COVID-19 vaccination for children	Yes	98 (43.75)
	No	126 (56.25)
Parent Attitudes About Childhood Vaccines (PACV) dichotomized	Non-hesitant (PACV Score < 21)	11 (7.56)
	Hesitant (PACV Score ≥ 21)	208 (92.44)

**Table 2:** Unadjusted crude analysis of the outcome of parents’ intention to give COVID-19 vaccine to their children and other covariates showing unadjusted odd ratios (ORs) and 95% confidence intervals (CI).

Variables	Category	Unadjusted OR (95%CI)	*p*-Value
Gender	Male	1	Ref.
	Female	1.94 (1.09–3.44)	0.020
Age	18–29	1	Ref.
	30–39	1.47 (0.50–4.30)	0.480
	40–49	0.63 (0.20–1.96)	0.430
	≥50	1.40 (0.31–6.33)	0.660
Relation to the child	Mother	1	Ref.
	Father	0.52 (0.29–0.92)	0.020
Education	High school and below	1	Ref.
	Undergraduate degree	5.45 (2.07–14.33)	0.001
	Postgraduate degree	2.62 (1.00–6.86)	0.040
Place of work	Government	1	Ref.
	Private	1.40 (0.69–2.80)	0.350
	Not employed	2.84 (1.20–6.73)	0.017
Work Sector	Health	1	Ref.
	Non-health	1.21 (0.71–2.06)	0.480
Insurance	Yes	1	Ref.
	No	1.32 (0.68–2.56)	0.397
Income	Not enough; took a loan and cannot pay back	1	Ref.
	Not enough; took a loan but can pay back	1.33 (0.42–4.30)	0.630
	Enough	0.97 (0.33–2.83)	0.980
	Enough and save	1.96 (0.53–7.31)	0.310
Older adults living within the same home	Yes	1	Ref.
	No	1.08 (0.62–2.00)	0.730
Family size	2	1	Ref.
	3–4	1.02 (0.30–3.43)	0.974
	≥5	0.83 (0.25–2.76)	0.757
Previous COVID-19 infection	Yes	1	Ref.
	No	0.36 (0.19–0.68)	0.002
	Not sure	0.46 (0.23–0.88)	0.020
Vaccine status	Does not want to take the vaccine	1	Ref.
	Took the first dose and is awaiting the second	0.13 (0.03–0.58)	0.007
	Took the first dose but does not want to take the second dose	0.31 (0.02–4.23)	0.380
	Took the first and second doses and is awaiting the booster dose	0.12 (0.08–0.37)	<0.001
	Took the first and second doses but did not want to take the booster dose	1.03 (0.23–4.59)	0.960
	Took the three doses	0.08 (0.02–0.30)	<0.001
	Wants to take the vaccine, but it is not scheduled yet	0.38 (0.08–1.84)	0.232
Children with chronic disease	No	1	Ref.
	Yes	1.8 (0.53–6.05)	0.337
Children intake of scheduled vaccines	Yes	1	Ref.
	No	1.55 (0.84–2.84)	0.158
Children intake for the influenza vaccine	Yes	1	Ref.
	No	1.65 (0.88–3.10)	0.117
	I do not know	0.75 (0.11–4.87)	0.763
A child with previous COVID-19 Infection	Yes	1	Ref.
	No	0.88 (0.40–1.94)	0.760
	I do not know	0.52 (0.20–1.34)	0.180
Parent Attitudes About Childhood Vaccines (PACV)	Non-hesitant (PACV Score < 21)	1	Ref.
	Hesitant (PACV Score ≥ 21)	11.20 (2.50–50.28)	0.002

**Table 3:** Multiple logistic regression analysis final model of the parents’ intention to give COVID-19 vaccine and various covariates with adjusted odds ratio (OR) and adjusted 95% confidence interval (CI).

Variables	Adjusted OR (95%) CI	*p*-Value
**Education**		
High school and below	1	Ref.
Undergraduate degree	3.58 (1.02–11.7)	0.045
Postgraduate degree	1.57 (0.40–6.05)	0.051
**Previous COVID-19 infection**		
Yes	1	Ref.
No	0.25 (0.10–0.58)	0.001
Not sure	0.20 (0.11–0.62)	0.002
**Vaccine status**		
Does not want to take the vaccine	1	Ref.
Took the first dose and is awaiting the second	0.07 (0.01–0.46)	0.005
Took the first dose but does not want to take the second dose	0.18 (0.01–3.53)	0.262
Took the first and second doses and is awaiting the booster dose	0.08 (0.02–0.35)	0.001
Took the first and second doses but did not want to take the booster dose	0.61 (0.10–3.75)	0.600
Took the three doses	0.04 (0.01–0.23)	<0.001
Wants to take the vaccine, but it is not scheduled yet	0.20 (0.03–1.42)	0.109
**Parent Attitudes About Childhood Vaccines (PACV)**		
Non-hesitant (PACV Score < 21)	1	Ref.
Hesitant (PACV Score ≥ 21)	10.80 (1.92–60.9)	0.007

**Table 4:** Descriptive statistics, reliability, and convergent validity of the Arabic version of the Parent Attitudes About Childhood Vaccines (PACV) scale.

Domain	Mean ± SD	Item-to-Score Correlation	*p*-Value
N = 224			
Total score	26.68 ± 4.46		
**Behavior**	3.89 ± 0.57		
Q1	1.95 ± 0.38	0.88	<0.001
Q2	1.94 ± 0.29	0.79	<0.001
Cronbach’s alpha	0.57		
**Attitude**	15.73 ± 3.28		
Q3	1.51 ± 0.76	0.60	<0.001
Q4	2.15 ± 0.83	0.41	<0.001
Q5	1.18 ± 0.46	0.66	<0.001
Q6	2.39 ± 0.75	0.02	0.811
Q7	1.76 ± 0.80	0.42	<0.001
Q11	1.21 ± 0.55	0.48	<0.001
Q12	1.53 ± 0.81	0.64	<0.001
Q13	1.38 ± 0.62	0.73	<0.001
Q14	1.25 ± 0.58	0.50	<0.001
Q15	1.36 ± 0.58	0.58	<0.001
Cronbach’s alpha	0.74		
**Safety and efficacy**	7.05 ± 2.14		
Q8	2.53 ± 0.77	0.84	<0.001
Q9	2.42 ± 0.91	0.89	<0.001
Q10	2.10 ± 0.70	0.79	<0.001
Cronbach’s alpha	0.82		
Overall Scale Cronbach’s alpha	0.80		

**Table 5:** Divergent validity of the Parental Attitudes About Childhood Vaccines (PACV) Tool.

Factors	Attitude	Safety and Efficacy	Behavior
**Attitude**	1	-	-
**Safety and efficacy**	0.51	1	-
**Behavior**	−0.093	0.032	1

**Table 6:** Factor loadings, subscales, and labeling of the Arabic version of the Parent Attitudes About Childhood Vaccines (PACV) scale.

Item	Attitude	Safety and Efficacy	Behavior	Uniqueness
Q1	0.10	0.05	0.57	0.66
Q2	−0.14	−0.10	0.60	0.59
Q3	0.55	0.09	−0.01	0.64
Q4	0.16	−0.06	−0.01	0.98
Q5	0.78	0.02	−0.07	0.39
Q6	−0.21	−0.18	−0.02	0.89
Q7	0.21	−0.09	−0.09	0.96
Q8	−0.02	0.80	−0.04	0.38
Q9	0.00	0.85	−0.03	0.27
Q10	0.24	0.51	0.07	0.55
Q11	0.42	0.04	−0.10	0.79
Q12	0.64	0.12	0.09	0.50
Q13	0.76	0.11	−0.03	0.32
Q14	0.67	−0.19	−0.02	0.64
Q15	0.61	0.05	0.11	0.66

## Data Availability

Data are available upon contacting the corresponding author (R.M.G.).

## Supplementary Materials

The following supporting information can be downloaded at: https://www.mdpi.com/article/10.3390/tropicalmed7090234/s1, Supplementary S1: The validated Arabic tool of Parents Attitude Toward Children Vaccination Tool; Supplementary S2: The backward translated version of the Parents Attitude Toward Children Vaccination Tool; Supplementary S3: Cognitive interview result; Supplementary S4: Data collection Tool.
