# Supplementary material for: Validation and Cultural Adaptation of the Parent Attitudes about Childhood Vaccines (PACV) Questionnaire in Arabic Language Widely Spoken in a Region with a High Prevalence of COVID-19 Vaccine Hesitancy
Source: Trop Med Infect Dis. 2022 Sep 8;7(9):234. doi: 10.3390/tropicalmed7090234 (PMC9504773; doi:10.3390/tropicalmed7090234)
Supplement: Supplementary file 1 [file tropicalmed-07-00234-s001.zip › Supplementary_S1.pdf]

Supplementary S1

Parent Attitudes about Childhood Vaccines (PACV) Scale in Arabic Language

1) هل سبق لك في أي وقت أن أجلت تطعيم طفلك لأسباب غير المرض أو الحساسية؟
2) هل سبق لك أن قررت الامتناع عن تطعيم طفلك لأسباب غير المرض أو الحساسية
3) إلى أي مدى أنت متأكد أن الالتزام بجرعات التطعيم الموصي بها مفيدة بالنسبة لطفلك
4) يتلقى الأطفال عدد أكبر من جرعات التطعيم اللازمة لهم
5) أعتقد أن التطعيمات تقي من الأمراض الشديدة
6) من الأفضل لطفلي أن يكتسب مناعة عن طريق الاصابة بالمرض بدلا من أخذ التطعيم.
7) من الأفضل أن يحصل الأطفال على عدد قليل من جرعات التطعيم في نفس الوقت.
8) الى أي مدى تشعر بالقلق من أن يسبب التطعيم عرض جانبي خطير لطفلك؟
9) الى أي مدى تشعر بالقلق أن تكون احدى تطعيمات الاطفال غير امنة؟
10) إلى أى مدى تشعر بالقلق أن التطعيم لن يقي من المرض؟
11) اذا رزقت بطفل في الوقت الحالي هل ترغب بأن يتلقى كل التطعيمات الموصى بها؟
12) عموما، الى أي درجة تعتبر نفسك متردد بشأن تطعيمات الأطفال؟
13) أثق بالمعلومات التي اتلقاها عن التطعيمات.
14) أنا قادر على مناقشة مخاوفي الخاصة بالتطعيم مع طبيب الأطفال.
15) اجمالا، الى أي مدى تثق في طبيب الأطفال ؟

In order to reuse this questionnaire, permission must be obtained from the corresponding authors: Ramy Mohamed Ghazy, E-mail: ramy_ghazy@alexu.edu.eg; Malik Sallam, Em-mail: malik.sallam@ju.edu.jo
