# Supplementary material for: Validation and Cultural Adaptation of the Parent Attitudes about Childhood Vaccines (PACV) Questionnaire in Arabic Language Widely Spoken in a Region with a High Prevalence of COVID-19 Vaccine Hesitancy
Source: Trop Med Infect Dis. 2022 Sep 8;7(9):234. doi: 10.3390/tropicalmed7090234 (PMC9504773; doi:10.3390/tropicalmed7090234)
Supplement: Supplementary file 1 [file tropicalmed-07-00234-s001.zip › Supplementary_S2_Backward_translation.pdf]

1. Have you ever delayed vaccinating your child for reasons other than illness or allergy?
2. Have you ever refrained from vaccinating your child for reasons other than illness or allergy?
3. To what extent are you certain that following the recommended vaccination schedule is good for your child?
4. Children receive more vaccination doses than is necessary for them.
5. I think/believe that vaccines prevent severe illnesses.
6. It is better for my child to acquire immunity by contracting the disease rather than getting a vaccine shot.
7. It is better that children receive fewer vaccines at the same time.
8. To what extent are you concerned that vaccination may cause your child to suffer a serious side effect?
9. To what extent are you concerned that any certain vaccination recommended for children is unsafe?
10. To what extent are you concerned that vaccines will not prevent disease?
11. If you had a child in the current period, would you like them to receive the recommended vaccinations?
12. Generally speaking, to what extent are you hesitant about childhood vaccinations?
13. I trust the information I receive about vaccines.
14. I can discuss my fears about vaccines with my child’s doctor.
15. All in all, how far do you trust your child’s doctor?
