# Supplementary material for: Validation and Cultural Adaptation of the Parent Attitudes about Childhood Vaccines (PACV) Questionnaire in Arabic Language Widely Spoken in a Region with a High Prevalence of COVID-19 Vaccine Hesitancy
Source: Trop Med Infect Dis. 2022 Sep 8;7(9):234. doi: 10.3390/tropicalmed7090234 (PMC9504773; doi:10.3390/tropicalmed7090234)
Supplement: Supplementary file 1 [file tropicalmed-07-00234-s001.zip › Supplementary_S3_Result_of_cognitive_interview.pdf]

Questions we tested

1) هل سبق لك في أي وقت أن أجلت تطعيم طفلك لأسباب غير المرض أو الحساسية؟
2) هل سبق لك أن قررت الامتناع عن تطعيم طفلك لأسباب غير المرض أو الحساسية
3) الى أي مدى أنت متأكد أن الالتزام بالجرعات الموصى بها مفيد بالنسبة لطفلك
4) يتلقى الأطفال عدد أكبر من الجرعات (جرعات التطعيم) اللازمة لهم
5) أعتقد أن التطعيمات تقي من الأمراض الشديدة
6) من الأفضل لطفلي أن يكتسب مناعة عن طريق الاصابة بالمرض عن (بدلا من) أخذ التطعيم.
7) من الأفضل أن يحصل الأطفال على عدد قليل من التطعيمات (جرعات التطعيم) في نفس الوقت.
8) الى أي مدى تشعر بالقلق من أن يسبب التطعيم عرض جانبي خطير لطفلك؟
9) الى أي مدى تشعر بالقلق أن تكون احدى تطعيمات الاطفال غير امنة؟
10) الى أن مدى تشعر بالقلق أن التطعيم لن يقي من المرض؟
11) اذا رزقت بطفل في الوقت الحالي هل ترغب بأن يتلقى كل التطعيمات الموصى بها؟
12) عموما، الى أي درجة تعتبر نفسك متردد بشأن تطعيمات الأطفال؟
13) أثق بالمعلومات التي اتلقاها عن التطعيمات.
14) أنا قادر على مناقشة مخاوفي الخاصة بالتطعيم مع طبيب طفلي (طبيب الاطفال) .
15) اجمالا، الى أي مدى تثق في طبيب طفلك (طبيب الاطفال) ؟

Pre-testing and cognitive interview

During cognitive interview, we recruited 16 participants (5 physicians, 3 nurses and 8 ordinary people). They were interviewed to determine if they got what is meant by each question.

We reportedthat:

- 9 participants are fully understanding the forward translated questions
- while 2 participants cannot understand what is meant by question number 4.“جرعات التطعيم”
- 1 participant commented on question 6“بدلا من اخذ التطعيم”
- 1 participant cannot understand question 7.“ جرعات التطعيم“
- 3 participants commented on question 14.

(They said ‘they have no concerns at earlier questions, so they have no issues to discuss with pediatrician.)

- 5 participants commented on question 15 as they have no pediatrician for their children.“طبيب الاطفال”
