# Supplementary material for: Validation and Cultural Adaptation of the Parent Attitudes about Childhood Vaccines (PACV) Questionnaire in Arabic Language Widely Spoken in a Region with a High Prevalence of COVID-19 Vaccine Hesitancy
Source: Trop Med Infect Dis. 2022 Sep 8;7(9):234. doi: 10.3390/tropicalmed7090234 (PMC9504773; doi:10.3390/tropicalmed7090234)
Supplement: Supplementary file 1 [file tropicalmed-07-00234-s001.zip › Supplementary_S4_Data_collection_tool.pdf]

مسح لتقييم مدى تقبل الآباء لتلقي لقاحات (تطعيمات) كورونا-19 بين أبنائهم ------------------------

---------------------يهديكم فريق الدراسة أطيب تحية، ويدعوكم فضلا للمشاركة في ملء الاستبيان المرفق. تهدف هذه الدراسة إلى تقييم مدى تقبل الآباء لتلقي لقاحات كورونا-19 بين أبنائهم الذين تتراوح أعمارهم بين 5 و17 سنة. ولمزيد من المعلومات لا تتردد في التواصل مع فريق البحث على الإيميل التالي: pacv.egypt@gmail.com هذا الاستبيان لن يطلب أي معلومات تدل على هويتك ولن يتم استخدام بياناتك سوى لغرض البحث.

* Required

Untitled Section

1. هل لديك أطفال تتراوح أعمارهم بين 5 سنوات إلى 17 سنة؟ *

Mark only one oval.

- ⬭ نعم
- ⬭ لا

2. ما هو عدد أطفالك الذين تتراوح أعمارهم بين 5 و 17 سنة؟ *

Mark only one oval.

- 1
- 2 *Skip to question 42*
- 3 *Skip to question 88*
- 4 *Skip to question 141*

البيانات الشخصية والصحية (خاصة بالآباء والأوصياء على الأطفال)

3. أذكر المحافظة التي تعيش بها *

Mark only one oval.

- الإسكندرية
- الإسماعيلية
- أسوان
- أسيوط
- الأقصر
- البحر الأحمر
- البحيرة
- بني سويف
- بورسعيد
- جنوب سيناء
- الجيزة
- الدقهلية
- دمياط
- سوهاج
- السويس
- الشرقية
- شمال سيناء
- الغربية
- الفيوم
- القاهرة
- القليوبية
- قنا

- كفر الشيخ
- مطروح
- المنوفية
- المنيا
- الوادي الجديد

4. الجنس/ النوع *

Mark only one oval.

- ذكر
- أنثى

5. العمر *

Mark only one oval.

- 18-29 عام
- 30-39 سنة
- 40-49 سنة
- 50-59 سنة
- أكثر من 60 سنة

6. صلة القرابة للطفل *

Mark only one oval.

- ( ) الأم
- ( ) الأب
- ( ) الجد/الجدة
- ( ) العم/ة أو الخال/ة
- ( ) غير ذلك

7. مستوى التعليم *

Mark only one oval.

- ( ) أمي لا يقرأ ولا يكتب
- ( ) يقرأ ويكتب
- ( ) ابتدائي
- ( ) اعدادي
- ( ) ثانوي
- ( ) جامعي
- ( ) دراسات عليا

8. محل العمل *

Mark only one oval.

- حكومي
- خاص
- لاأعمل

9. نوع العمل *

Mark only one oval.

- أعمل من خلال المنزل
- أعمل جزئيا (أذهب الي العمل 3 أو 4 مرات في الأسبوع)
- أذهب إلي العمل يوميا
- لاأعمل

10. هل تعمل بالمجال الصحي؟ *

Mark only one oval.

- نعم
- لا

11. هل لديك تأمين صحي؟ *

Mark only one oval.

- ( ) تأمين صحي حكومي
- ( ) تأمين صحي خاص
- ( ) بدون تـأمين صحي

12. الدخل الشهري *

Mark only one oval.

- ( ) لا يكفي ويستدين ولا يستطيع الوفاء بالدين
- ( ) لا يكفي ويستدين ويستطيع الوفاء بالدين
- ( ) يكفي
- ( ) يكفي ويدخر

13. هل يعيش أحد الوالدين أو الأقارب من كبار السن معك في نفس المنزل؟ *

Mark only one oval.

- ( ) نعم
- ( ) لا

14. ما هو عدد الأفراد الذين يعيشون بداخل منزلك؟ *

Mark only one oval.

- فردين
- 3 أفراد
- 4 أفراد
- 5 أفراد
- أكثر من 5 أفراد

15. هل تأكدت إصابتك بفيروس كورونا-19 من قبل؟ *

Mark only one oval.

- نعم
- لا
- غير متأكد

16. ما هو موقفك من لقاح كورونا-19؟ *

Mark only one oval.

- ( ) أرفض تلقي اللقاح (التطعيم)
- ( ) تلقيت الجرعة الأولي وبانتظار الجرعة الثانية
- ( ) تلقيت الجرعة الأولي ولن أتلقي الجرعة الثانية
- ( ) تلقيت الجرعة الأولي والثانية وفي انتظار الجرعة التنشيطية
- ( ) تلقيت الجرعة الأولي والثانية ولن أكمل الجرعة التنشيطية
- ( ) أتممت الجرعات (الأولي والثانية والتنشيطية)
- ( ) أوافق علي تلقي اللقاح وبانتظار تلقي الجرعات

17. ما هو سبب عدم استكمال جرعات اللقاح المضاد لفيروس كورونا-19؟ *

Check all that apply.

- [ ] لأن التطعيم غير متوفر
- [ ] لن استكمل الجرعات
- [ ] لأن ميعاد الجرعة الثانية من اللقاح لم يحن موعده بعد
- [ ] لا ينطبق

Other: [ ] ______________________

18. إن كنت تعاني من أمراض مزمنة, حدد تلك الأمراض *

Check all that apply.

- [ ] لا أعاني من أمراض مزمنة
- [ ] ضغط
- [ ] سكر
- [ ] قلب
- [ ] أمراض صدرية
- [ ] أمراض في الكلى والكبد
- [ ] أمراض روماتيزمية ومناعية
- [ ] أمراض نفسية وعصبية
- [ ] إعاقة حركية
- [ ] فقدان جزئي للسمع والبصر

Other: [ ] ____________________

بيانات خاصة بطفلك

19. ما هو عمر طفلك؟ *

____

20. ما هو نوع/جنس طفلك؟ *

Mark only one oval.

- ذكر
- أنثى

21. هل سبق وأن تم تشخيص طفلك بالإصابة بفيروس كورونا-19؟ *

Mark only one oval.

- نعم
- لا
- غير متأكد

22. هل يعاني طفلك من أي أمراض مزمنة؟ (حدد تلك الأمراض) *

Check all that apply.

- [ ] لا يعاني أبني من أي أمراض مزمنة
- [ ] سكر
- [ ] ضغط
- [ ] قلب
- [ ] أمراض صدرية
- [ ] أمراض الكلى والكبد
- [ ] أمراض روماتيزمية ومناعية
- [ ] أمراض نفسية وعصبية
- [ ] إعاقات حركية
- [ ] فقدان جزئي للسمع أو البصر

23. هل تلقى طفلك التطعيمات (اللقاحات) الإجبارية للأطفال من قبل وزارة الصحة المصرية؟ *

Mark only one oval.

- ( ) نعم
- ( ) لا
- ( ) غير متأكد

24. هل تلقى طفلك تطعيم الانفلونزا الموسمية في خلال السنتين الماضيتين؟ *

Mark only one oval.

- نعم
- لا
- غير متأكد

25. ما هو موقفك تجاه تطعيم طفلك بتطعيم (لقاح) الكورونا-19؟ *

Mark only one oval.

- بالفعل تلقى طفلي تطعيم (لقاح) الكورونا-19
- أوافق على تطعيم طفلي, ولكن تطعيم (لقاح) الكورونا-19 غير متوفر
- أرفض تطعيم طفلي بتطعيم (لقاح) كورونا-19

26. إذا أتيح لك الاختيار، هل ترغب في تطعيم طفلك بنوع محدد من اللقاحات المتوفرة ضد فيروس كورونا-19؟ *

Check all that apply.

- [ ] سينوفارم
- [ ] سينوفاك
- [ ] استرازينيكا
- [ ] سبوتنيك
- [ ] جونسون اند جونسون
- [ ] فايزر
- [ ] مودرنا
- [ ] أرفض تطعيم طفلي
- [ ] لا أعلم

مقياس اتجاهات الآباء عن التطعيمات لأطفالهم

الرجاء اختيار إجابة واحدة

27. هل سبق لك في أي وقت أن أجلت تطعيم طفلك لأسباب غير المرض أو الحساسية؟ *

Mark only one oval.

- ( ) نعم
- ( ) لا
- ( ) لا أعلم

28. هل سبق لك أن قررت الامتناع عن تطعيم طفلك لأسباب غير المرض أو الحساسية؟ *

Mark only one oval.

- نعم
- لا
- لا أعلم

29. إلى أي مدى أنت متأكد أن الالتزام بجرعات التطعيم الموصي بها مفيدة بالنسبة لطفلك *

Mark only one oval.

	1	2	3	4	5	6	7	8	9	10	
غير متأكد علي الإطلاق											متأكد بشدة

30. يتلقى الأطفال عدد أكبر من جرعات التطعيم اللازمة لهم *

Mark only one oval.

- أوافق بشدة
- أوافق
- غير متأكد
- لا أوافق
- لا أوافق بشدة

31. أعتقد أن التطعيمات تقي من الأمراض الشديدة *

Mark only one oval.

- أوافق بشدة
- أوافق
- غير متأكد
- لا أوافق
- لا أوافق بشدة

32. من الأفضل لطفلي أن يكتسب مناعة عن طريق الإصابة بالمرض بدلا من أخذ التطعيم *

Mark only one oval.

- أوافق بشدة
- أوافق
- غير متأكد
- لا أوافق
- لا أوافق بشدة

33. من الأفضل أن يحصل الأطفال على عدد قليل من جرعات التطعيم في نفس الوقت *

Mark only one oval.

- أوافق بشدة
- أوافق
- غير متأكد
- لا أوافق
- لا أوافق بشدة

34. إلى أي مدى تشعر بالقلق من أن يْسبب التطعيم عرض جانبي خطير لطفلك؟ *

Mark only one oval.

- أنا لست قلق أبدا تجاه ذلك
- أنا لست قلق تجاه ذلك
- غير متأكد
- أنا قلق قليلا تجاه ذلك
- أنا قلق كثيرا تجاه ذلك

35. إلى أي مدى تشعر بالقلق أن تكون احدى تطعيمات الاطفال غير امنة؟ *

Mark only one oval.

- أنا لست قلق أبدا تجاه ذلك
- أنا لست قلق تجاه ذلك
- أنا قلق قليلا تجاه ذلك
- أنا قلق كثيرا تجاه ذلك

36. إلى أي مدى تشعر بالقلق أن التطعيم لن يقي من المرض؟ *

Mark only one oval.

- أنا لست قلق أبدا تجاه ذلك
- أنا لست قلق تجاه ذلك
- غير متأكد
- أنا قلق قليلا تجاه ذلك
- أنا قلق كثيرا تجاه ذلك

37. إذا رزقت بطفل في الوقت الحالي هل ترغب بأن يتلقى كل التطعيمات الموصى بها؟ *

Mark only one oval.

- نعم
- لا
- لا أعلم

38. عموما، الى أي درجة تعتبر نفسك متردد بشأن تطعيمات الأطفال؟ *

Mark only one oval.

- لست متردد أبدا
- لست متردد
- غير متأكد
- متردد
- متردد جدا

39. أثق بالمعلومات التي اتلقاها عن التطعيمات *

Mark only one oval.

- أوافق بشدة
- أوافق
- غير متأكد
- لا أوافق
- لا أوافق بشدة

40. أنا قادر على مناقشة مخاوفي الخاصة بالتطعيم مع طبيب طفلي *

Mark only one oval.

- أوافق بشدة
- أوافق
- غير متأكد
- لا أوافق
- لا أوافق بشدة

41. إجمالا، الى أي مدى تثق في طبيب طفلك؟ *

Mark only one oval.

	1	2	3	4	5	6	7	8	9	10	
لا أثق علي الإطلاق	◯	◯	◯	◯	◯	◯	◯	◯	◯	◯	أثق بشدة

نشكر تعاونكم وتقبلوا تحيات فريق البحث

البيانات الشخصية والصحية (خاصة بالآباء والأوصياء على الأطفال)

42. أذكر المحافظة التي تعيش بها *

Mark only one oval.

- الإسكندرية
- الإسماعيلية
- أسوان
- أسيوط
- الأقصر
- البحر الأحمر
- البحيرة
- بني سويف
- بورسعيد
- جنوب سيناء
- الجيزة
- الدقهلية
- دمياط
- سوهاج
- السويس
- الشرقية
- شمال سيناء
- الغربية
- الفيوم
- القاهرة
- القليوبية
- قنا

- كفر الشيخ
- مطروح
- المنوفية
- المنيا
- الوادي الجديد

43. الجنس/ النوع *

Mark only one oval.

- ذكر
- أنثى

44. العمر *

Mark only one oval.

- 18-29 عام
- 30-39 سنة
- 40-49 سنة
- 50-59 سنة
- أكثر من 60 سنة

45. صلة القرابة للطفل *

Mark only one oval.

- الأم
- الأب
- الجد/الجدة
- العم/ة أو الخال/ة
- غير ذلك

46. مستوى التعليم *

Mark only one oval.

- أمي لا يقرأ ولا يكتب
- يقرأ ويكتب
- ابتدائي
- اعدادي
- ثانوي
- جامعي
- دراسات عليا

47. محل العمل *

Mark only one oval.

- حكومي
- خاص
- لاأعمل

48. نوع العمل *

Mark only one oval.

- أعمل من خلال المنزل
- أعمل جزئيا (أذهب الي العمل 3 أو 4 مرات في الأسبوع)
- أذهب إلي العمل يوميا
- لاأعمل

49. هل تعمل بالمجال الصحي؟ *

Mark only one oval.

- نعم
- لا

50. هل لديك تأمين صحي؟ *

Mark only one oval.

- تأمين صحي حكومي
- تأمين صحي خاص
- بدون تـأمين صحي

51. الدخل الشهري *

Mark only one oval.

- لا يكفي ويستدين ولا يستطيع الوفاء بالدين
- لا يكفي ويستدين ويستطيع الوفاء بالدين
- يكفي
- يكفي ويدخر

52. هل يعيش أحد الوالدين أو الأقارب من كبار السن معك في نفس المنزل؟ *

Mark only one oval.

- نعم
- لا

53. ما هو عدد الأفراد الذين يعيشون بداخل منزلك؟ *

Mark only one oval.

- فردين
- 3 أفراد
- 4 أفراد
- 5 أفراد
- أكثر من 5 أفراد

54. هل تأكدت إصابتك بفيروس كورونا-19 من قبل؟ *

Mark only one oval.

- نعم
- لا
- غير متأكد

55. ما هو موقفك من لقاح كورونا-19؟ *

Mark only one oval.

- ( ) أرفض تلقي اللقاح (التطعيم)
- ( ) تلقيت الجرعة الأولي وبانتظار الجرعة الثانية
- ( ) تلقيت الجرعة الأولي ولن أتلقي الجرعة الثانية
- ( ) تلقيت الجرعة الأولي والثانية وفي انتظار الجرعة التنشيطية
- ( ) تلقيت الجرعة الأولي والثانية ولن أكمل الجرعة التنشيطية
- ( ) أتممت الجرعات (الأولي والثانية والتنشيطية)
- ( ) أوافق علي تلقي اللقاح وبانتظار تلقي الجرعات

56. ما هو سبب عدم استكمال جرعات اللقاح المضاد لفيروس كورونا-19؟ *

Check all that apply.

- [ ] لأن التطعيم غير متوفر
- [ ] لن استكمل الجرعات
- [ ] لأن ميعاد الجرعة الثانية من اللقاح لم يحن موعده بعد
- [ ] لا ينطبق

Other: [ ] ____________________

57. إن كنت تعاني من أمراض مزمنة, حدد تلك الأمراض *

Check all that apply.

- [ ] لا أعاني من أمراض مزمنة
- [ ] ضغط
- [ ] سكر
- [ ] قلب
- [ ] أمراض صدرية
- [ ] أمراض في الكلى والكبد
- [ ] أمراض روماتيزمية ومناعية
- [ ] أمراض نفسية وعصبية
- [ ] إعاقة حركية
- [ ] فقدان جزئي للسمع والبصر
- [ ] Other: ____________________

البيانات الخاصة بالطفل الأول

58. ما هو عمر الطفل الأول؟ *

____

59. ما هو جنس/نوع الطفل الأول؟ *

Mark only one oval.

- ذكر
- انثى

60. هل سبق وأن تم تشخيص طفلك الأول بالإصابة بفيروس كورونا-19؟ *

Mark only one oval.

- نعم
- لا
- غير متأكد

61. هل يعاني طفلك الأول من أي أمراض مزمنة؟ (حدد تلك الأمراض) *

Check all that apply.

- [ ] لا يعاني أبني من أي أمراض مزمنة
- [ ] سكر
- [ ] ضغط
- [ ] قلب
- [ ] أمراض صدرية
- [ ] أمراض الكلى والكبد
- [ ] أمراض روماتيزمية ومناعية
- [ ] أمراض نفسية وعصبية
- [ ] إعاقات حركية
- [ ] فقدان جزئي للسمع أو البصر

62. هل تلقى طفلك الأول التطعيمات (اللقاحات) الإجبارية للأطفال من قبل وزارة الصحة المصرية؟ *

Mark only one oval.

- ( ) نعم
- ( ) لا
- ( ) غير متأكد

63. هل تلقى طفلك الأول تطعيم الانفلونزا الموسمية في خلال السنتين الماضيتين؟ *

Mark only one oval.

- نعم
- لا
- غير متأكد

64. ما هو موقفك تجاه تطعيم طفلك بتطعيم (لقاح) الكورونا-19؟ *

Mark only one oval.

- بالفعل تلقى طفلي تطعيم (لقاح) الكورونا-19
- أوافق على تطعيم طفلي ولكن تطعيم (لقاح) الكورونا-19 غيرمتوفر
- أرفض تطعيم طفلي بتطعيم (لقاح) كورونا-19

البيانات الخاصة بالطفل الثاني

65. ما هو عمر الطفل الثاني؟ *

66. ما هو جنس/نوع الطفل الثاني؟ *

Mark only one oval.

- ذكر
- انثى

67. هل سبق وأن تم تشخيص طفلك الثاني بالإصابة بفيروس كورونا-19؟ *

Mark only one oval.

- نعم
- لا
- غير متأكد

68. (هل يعاني طفلك الثاني من أي أمراض مزمنة؟ (حدد تلك الأمراض *

Check all that apply.

- [ ] لا يعاني أبني من أي أمراض مزمنة
- [ ] سكر
- [ ] ضغط
- [ ] قلب
- [ ] أمراض صدرية
- [ ] أمراض الكلى والكبد
- [ ] أمراض روماتيزمية ومناعية
- [ ] أمراض نفسية وعصبية
- [ ] إعاقات حركية
- [ ] فقدان جزئي للسمع أو البصر

69. هل تلقى طفلك الثاني التطعيمات (اللقاحات) الإجبارية للأطفال من قبل وزارة الصحة المصرية؟ *

Mark only one oval.

- ( ) نعم
- ( ) لا
- ( ) غير متأكد

70. هل تلقى طفلك الثاني تطعيم الانفلونزا الموسمية في خلال السنتين الماضيتين؟ *

Mark only one oval.

- نعم
- لا
- غير متأكد

71. ما هو موقفك تجاه تطعيم طفلك بتطعيم (لقاح) الكورونا-19؟ *

Mark only one oval.

- بالفعل تلقى طفلي تطعيم (لقاح) الكورونا-19
- أوافق على تطعيم طفلي ولكن تطعيم (لقاح) الكورونا-19 غيرمتوفر
- أرفض تطعيم طفلي بتطعيم (لقاح) كورونا-19

72. إذا أتيح لك الاختيار، هل ترغب في تطعيم أطفالك نوع محدد من اللقاحات المتوفرة ضد فيروس كورونا-19؟ *

Check all that apply.

- [ ] سينوفارم
- [ ] سينوفاك
- [ ] استرازينيكا
- [ ] سبوتنيك
- [ ] جونسون اند جونسون
- [ ] فايزر
- [ ] مودرنا
- [ ] أرفض تطعيم أطفالي
- [ ] لا أعلم

الرجاء اختيار إجابة واحدة

مقياس اتجاهات الآباء عن التطعيمات لأطفالهم

73. هل سبق لك في أي وقت أن أجلت تطعيم طفلك لأسباب غير المرض أو الحساسية؟ *

Mark only one oval.

- ( ) نعم
- ( ) لا
- ( ) لا أعلم

74. هل سبق لك أن قررت الامتناع عن تطعيم طفلك لأسباب غير المرض أو الحساسية؟ *

Mark only one oval.

- نعم
- لا
- لا أعلم

75. إلى أي مدى أنت متأكد أن الالتزام بجرعات التطعيم الموصي بها مفيدة بالنسبة لطفلك *

Mark only one oval.

	1	2	3	4	5	6	7	8	9	10	
غير متأكد علي الإطلاق											متأكد بشدة

76. يتلقى الأطفال عدد أكبر من جرعات التطعيم اللازمة لهم *

Mark only one oval.

- أوافق بشدة
- أوافق
- غير متأكد
- لا أوافق
- لا أوافق بشدة

77. أعتقد أن التطعيمات تقي من الأمراض الشديدة *

Mark only one oval.

- أوافق بشدة
- أوافق
- غير متأكد
- لا أوافق
- لا أوافق بشدة

78. من الأفضل لطفلي أن يكتسب مناعة عن طريق الإصابة بالمرض بدلا من أخذ التطعيم *

Mark only one oval.

- أوافق بشدة
- أوافق
- غير متأكد
- لا أوافق
- لا أوافق بشدة

79. من الأفضل أن يحصل الأطفال على عدد قليل من جرعات التطعيم في نفس الوقت *

Mark only one oval.

- أوافق بشدة
- أوافق
- غير متأكد
- لا أوافق
- لا أوافق بشدة

80. إلى أي مدى تشعر بالقلق من أن يسبب التطعيم عرض جانبي خطير لطفلك؟ *

Mark only one oval.

- أنا لست قلق أبدا تجاه ذلك
- أنا لست قلق تجاه ذلك
- غير متأكد
- أنا قلق قليلا تجاه ذلك
- أنا قلق كثيرا تجاه ذلك

81. إلى أي مدى تشعر بالقلق أن تكون احدى تطعيمات الاطفال غير امنة؟ *

Mark only one oval.

- أنا لست قلق أبدا تجاه ذلك
- أنا لست قلق تجاه ذلك
- أنا قلق قليلا تجاه ذلك
- أنا قلق كثيرا تجاه ذلك

82. إلى أي مدى تشعر بالقلق أن التطعيم لن يقي من المرض؟ *

Mark only one oval.

- أنا لست قلق أبدا تجاه ذلك
- أنا لست قلق تجاه ذلك
- غير متأكد
- أنا قلق قليلا تجاه ذلك
- أنا قلق كثيرا تجاه ذلك

83. إذا رزقت بطفل في الوقت الحالي هل ترغب بأن يتلقى كل التطعيمات الموصى بها؟ *

Mark only one oval.

- نعم
- لا
- لا أعلم

84. عموما، الى أي درجة تعتبر نفسك متردد بشأن تطعيمات الأطفال؟ *

Mark only one oval.

- لست متردد أبدا
- لست متردد
- غير متأكد
- متردد
- متردد جدا

85. أثق بالمعلومات التي اتلقاها عن التطعيمات *

Mark only one oval.

- أوافق بشدة
- أوافق
- غير متأكد
- لا أوافق
- لا أوافق بشدة

86. أنا قادر على مناقشة مخاوفي الخاصة بالتطعيم مع طبيب طفلي *

Mark only one oval.

- أوافق بشدة
- أوافق
- غير متأكد
- لا أوافق
- لا أوافق بشدة

87. إجمالا، الى أي مدى تثق في طبيب طفلك؟ *

Mark only one oval.

	1	2	3	4	5	6	7	8	9	10	
لا أثق علي الإطلاق	◯	◯	◯	◯	◯	◯	◯	◯	◯	◯	أثق بشدة

نشكر تعاونكم وتقبلوا تحيات فريق البحث

البيانات الشخصية والصحية (خاصة بالآباء والأوصياء على الأطفال)

88. أذكر المحافظة التي تعيش بها *

Mark only one oval.

- الإسكندرية
- الإسماعيلية
- أسوان
- أسيوط
- الأقصر
- البحر الأحمر
- البحيرة
- بني سويف
- بورسعيد
- جنوب سيناء
- الجيزة
- الدقهلية
- دمياط
- سوهاج
- السويس
- الشرقية
- شمال سيناء
- الغربية
- الفيوم
- القاهرة
- القليوبية
- قنا

- كفر الشيخ
- مطروح
- المنوفية
- المنيا
- الوادي الجديد

89. الجنس/ النوع *

Mark only one oval.

- ذكر
- أنثى

90. العمر *

Mark only one oval.

- 18-29 عام
- 30-39 سنة
- 40-49 سنة
- 50-59 سنة
- أكثر من 60 سنة

91. صلة القرابة للطفل *

Mark only one oval.

- الأم
- الأب
- الجد/الجدة
- العم/ة أو الخال/ة
- غير ذلك

92. مستوى التعليم *

Mark only one oval.

- أمي لا يقرأ ولا يكتب
- يقرأ ويكتب
- ابتدائي
- اعدادي
- ثانوي
- جامعي
- دراسات عليا

93. محل العمل *

Mark only one oval.

- حكومي
- خاص
- لاأعمل

94. نوع العمل *

Mark only one oval.

- أعمل من خلال المنزل
- أعمل جزئيا (أذهب الي العمل 3 أو 4 مرات في الأسبوع)
- أذهب إلي العمل يوميا
- لاأعمل

95. هل تعمل بالمجال الصحي؟ *

Mark only one oval.

- نعم
- لا

96. هل لديك تأمين صحي؟ *

Mark only one oval.

- تأمين صحي حكومي
- تأمين صحي خاص
- بدون تـأمين صحي

97. الدخل الشهري *

Mark only one oval.

- لا يكفي ويستدين ولا يستطيع الوفاء بالدين
- لا يكفي ويستدين ويستطيع الوفاء بالدين
- يكفي
- يكفي ويدخر

98. هل يعيش أحد الوالدين أو الأقارب من كبار السن معك في نفس المنزل؟ *

Mark only one oval.

- نعم
- لا

99. ما هو عدد الأفراد الذين يعيشون بداخل منزلك؟ *

Mark only one oval.

- فردين
- 3 أفراد
- 4 أفراد
- 5 أفراد
- أكثر من 5 أفراد

100. هل تأكدت إصابتك بفيروس كورونا-19 من قبل؟ *

Mark only one oval.

- نعم
- لا
- غير متأكد

101. ما هو موقفك من لقاح كورونا-19؟ *

Mark only one oval.

- ( ) أرفض تلقي اللقاح (التطعيم)
- ( ) تلقيت الجرعة الأولي وبانتظار الجرعة الثانية
- ( ) تلقيت الجرعة الأولي ولن أتلقي الجرعة الثانية
- ( ) تلقيت الجرعة الأولي والثانية وفي انتظار الجرعة التنشيطية
- ( ) تلقيت الجرعة الأولي والثانية ولن أكمل الجرعة التنشيطية
- ( ) أتممت الجرعات (الأولي والثانية والتنشيطية)
- ( ) أوافق علي تلقي اللقاح وبانتظار تلقي الجرعات

102. ما هو سبب عدم استكمال جرعات اللقاح المضاد لفيروس كورونا-19؟ *

Check all that apply.

- [ ] لأن التطعيم غير متوفر
- [ ] لن استكمل الجرعات
- [ ] لأن ميعاد الجرعة الثانية من اللقاح لم يحن موعده بعد
- [ ] لا ينطبق

Other: [ ] ______________________

103. إن كنت تعاني من أمراض مزمنة, حدد تلك الأمراض *

Check all that apply.

- [ ] لا أعاني من أمراض مزمنة
- [ ] ضغط
- [ ] سكر
- [ ] قلب
- [ ] أمراض صدرية
- [ ] أمراض في الكلى والكبد
- [ ] أمراض روماتيزمية ومناعية
- [ ] أمراض نفسية وعصبية
- [ ] إعاقة حركية
- [ ] فقدان جزئي للسمع والبصر
- [ ] Other: ____________________

البيانات الخاصة بالطفل الأول

104. ما هو عمر الطفل الأول؟ *

____

105. ما هو جنس/نوع الطفل الأول؟ *

Mark only one oval.

- ذكر
- انثى

106. هل سبق وأن تم تشخيص طفلك الأول بالإصابة بفيروس كورونا-19؟ *

Mark only one oval.

- نعم
- لا
- غير متأكد

107. هل يعاني طفلك الأول من أي أمراض مزمنة؟ (حدد تلك الأمراض) *

Check all that apply.

- [ ] لا يعاني أبني من أي أمراض مزمنة
- [ ] سكر
- [ ] ضغط
- [ ] قلب
- [ ] أمراض صدرية
- [ ] أمراض الكلى والكبد
- [ ] أمراض روماتيزمية ومناعية
- [ ] أمراض نفسية وعصبية
- [ ] إعاقات حركية
- [ ] فقدان جزئي للسمع أو البصر

108. هل تلقى طفلك الأول التطعيمات (اللقاحات) الإجبارية للأطفال من قبل وزارة الصحة المصرية؟ *

Mark only one oval.

- ( ) نعم
- ( ) لا
- ( ) غير متأكد

109. هل تلقى طفلك الأول تطعيم الانفلونزا الموسمية في خلال السنتين الماضيتين؟ *

Mark only one oval.

- نعم
- لا
- غير متأكد

110. ما هو موقفك تجاه تطعيم طفلك بتطعيم (لقاح) الكورونا-19؟ *

Mark only one oval.

- بالفعل تلقى طفلي تطعيم (لقاح) الكورونا-19
- أوافق على تطعيم طفلي ولكن تطعيم (لقاح) الكورونا-19 غيرمتوفر
- أرفض تطعيم طفلي بتطعيم (لقاح) كورونا-19

البيانات الخاصة بالطفل الثاني

111. ما هو عمر الطفل الثاني؟ *

112. ما هو جنس/نوع الطفل الثاني؟ *

Mark only one oval.

- ذكر
- انثى

113. هل سبق وأن تم تشخيص طفلك الثاني بالإصابة بفيروس كورونا-19؟ *

Mark only one oval.

- نعم
- لا
- غير متأكد

114. هل يعاني طفلك الثاني من أي أمراض مزمنة؟ (حدد تلك الأمراض) *

Check all that apply.

- [ ] لا يعاني أبني من أي أمراض مزمنة
- [ ] سكر
- [ ] ضغط
- [ ] قلب
- [ ] أمراض صدرية
- [ ] أمراض الكلى والكبد
- [ ] أمراض روماتيزمية ومناعية
- [ ] أمراض نفسية وعصبية
- [ ] إعاقات حركية
- [ ] فقدان جزئي للسمع أو البصر

115. هل تلقى طفلك الثاني التطعيمات (اللقاحات) الإجبارية للأطفال من قبل وزارة الصحة المصرية؟ *

Mark only one oval.

- ( ) نعم
- ( ) لا
- ( ) غير متأكد

116. هل تلقى طفلك الثاني تطعيم الانفلونزا الموسمية في خلال السنتين الماضيتين؟ *

Mark only one oval.

- نعم
- لا
- غير متأكد

117. ما هو موقفك تجاه تطعيم طفلك بتطعيم (لقاح) الكورونا-19؟ *

Mark only one oval.

- بالفعل تلقى طفلي تطعيم (لقاح) الكورونا-19
- أوافق على تطعيم طفلي ولكن تطعيم (لقاح) الكورونا-19 غيرمتوفر
- أرفض تطعيم طفلي بتطعيم (لقاح) كورونا-19

البيانات الخاصة بالطفل الثالث

118. ما هو عمر الطفل الثالث؟ *

119. ما هو جنس/نوع الطفل الثالث؟ *

Mark only one oval.

- ( ) ذكر
- ( ) انثى

120. هل سبق وأن تم تشخيص طفلك الثالث بالإصابة بفيروس كورونا-19؟ *

Mark only one oval.

- ( ) نعم
- ( ) لا
- ( ) غير متأكد

121. هل يعاني طفلك الثالث من أي أمراض مزمنة؟ (حدد تلك الأمراض) *

Check all that apply.

- [ ] لا يعاني أبني من أي أمراض مزمنة
- [ ] سكر
- [ ] ضغط
- [ ] قلب
- [ ] أمراض صدرية
- [ ] أمراض الكلى والكبد
- [ ] أمراض روماتيزمية ومناعية
- [ ] أمراض نفسية وعصبية
- [ ] إعاقات حركية
- [ ] فقدان جزئي للسمع أو البصر

122. هل تلقى طفلك الثالث التطعيمات (اللقاحات) الإجبارية للأطفال من قبل وزارة الصحة المصرية؟ *

Mark only one oval.

- ( ) نعم
- ( ) لا
- ( ) غير متأكد

123. هل تلقى طفلك الثالث تطعيم الانفلونزا الموسمية في خلال السنتين الماضيتين؟ *

Mark only one oval.

- نعم
- لا
- غير متأكد

124. ما هو موقفك تجاه تطعيم طفلك بتطعيم (لقاح) الكورونا-19؟ *

Mark only one oval.

- بالفعل تلقى طفلي تطعيم (لقاح) الكورونا-19
- أوافق على تطعيم طفلي ولكن تطعيم (لقاح) الكورونا-19 غيرمتوفر
- أرفض تطعيم طفلي بتطعيم (لقاح) كورونا-19

125. إذا أتيح لك الاختيار، هل ترغب في تطعيم أطفالك نوع محدد من اللقاحات المتوفرة ضد فيروس كورونا-19؟ *

Check all that apply.

- [ ] سينوفارم
- [ ] سينوفاك
- [ ] استرازينيكا
- [ ] سبوتنيك
- [ ] جونسون اند جونسون
- [ ] فايزر
- [ ] مودرنا
- [ ] أرفض تطعيم أطفالي
- [ ] لا أعلم

مقياس اتجاهات الآباء عن التطعيمات لأطفالهم

الرجاء اختيار إجابة واحدة

126. هل سبق لك في أي وقت أن أجلت تطعيم طفلك لأسباب غير المرض أو الحساسية؟ *

Mark only one oval.

- ( ) نعم
- ( ) لا
- ( ) لا أعلم

127. هل سبق لك أن قررت الامتناع عن تطعيم طفلك لأسباب غير المرض أو الحساسية؟ *

Mark only one oval.

- نعم
- لا
- لا أعلم

128. إلى أي مدى أنت متأكد أن الالتزام بجرعات التطعيم الموصي بها مفيدة بالنسبة لطفلك *

Mark only one oval.

	1	2	3	4	5	6	7	8	9	10	
غير متأكد علي الإطلاق											متأكد بشدة

129. يتلقى الأطفال عدد أكبر من جرعات التطعيم اللازمة لهم *

Mark only one oval.

- أوافق بشدة
- أوافق
- غير متأكد
- لا أوافق
- لا أوافق بشدة

130. أعتقد أن التطعيمات تقي من الأمراض الشديدة *

Mark only one oval.

- أوافق بشدة
- أوافق
- غير متأكد
- لا أوافق
- لا أوافق بشدة

131. من الأفضل لطفلي أن يكتسب مناعة عن طريق الإصابة بالمرض بدلا من أخذ التطعيم *

Mark only one oval.

- أوافق بشدة
- أوافق
- غير متأكد
- لا أوافق
- لا أوافق بشدة

132. من الأفضل أن يحصل الأطفال على عدد قليل من جرعات التطعيم في نفس الوقت *

Mark only one oval.

- أوافق بشدة
- أوافق
- غير متأكد
- لا أوافق
- لا أوافق بشدة

133. إلى أي مدى تشعر بالقلق من أن يسبب التطعيم عرض جانبي خطير لطفلك؟ *

Mark only one oval.

- أنا لست قلق أبدا تجاه ذلك
- أنا لست قلق تجاه ذلك
- غير متأكد
- أنا قلق قليلا تجاه ذلك
- أنا قلق كثيرا تجاه ذلك

134. إلى أي مدى تشعر بالقلق أن تكون احدى تطعيمات الاطفال غير امنة؟ *

Mark only one oval.

- أنا لست قلق أبدا تجاه ذلك
- أنا لست قلق تجاه ذلك
- أنا قلق قليلا تجاه ذلك
- أنا قلق كثيرا تجاه ذلك

135. إلى أي مدى تشعر بالقلق أن التطعيم لن يقي من المرض؟ *

Mark only one oval.

- أنا لست قلق أبدا تجاه ذلك
- أنا لست قلق تجاه ذلك
- غير متأكد
- أنا قلق قليلا تجاه ذلك
- أنا قلق كثيرا تجاه ذلك

136. إذا رزقت بطفل في الوقت الحالي هل ترغب بأن يتلقى كل التطعيمات الموصى بها؟ *

Mark only one oval.

- نعم
- لا
- لا أعلم

137. عموما، الى أي درجة تعتبر نفسك متردد بشأن تطعيمات الأطفال؟ *

Mark only one oval.

- لست متردد أبدا
- لست متردد
- غير متأكد
- متردد
- متردد جدا

138. أثق بالمعلومات التي اتلقاها عن التطعيمات *

Mark only one oval.

- أوافق بشدة
- أوافق
- غير متأكد
- لا أوافق
- لا أوافق بشدة

139. أنا قادر على مناقشة مخاوفي الخاصة بالتطعيم مع طبيب طفلي *

Mark only one oval.

- أوافق بشدة
- أوافق
- غير متأكد
- لا أوافق
- لا أوافق بشدة

140. إجمالا، الى أي مدى تثق في طبيب طفلك؟ *

Mark only one oval.

	1	2	3	4	5	6	7	8	9	10	
لا أثق علي الإطلاق											أثق بشدة

نشكر تعاونكم وتقبلوا تحيات فريق البحث

(البيانات الشخصية والصحية (خاصة بالآباء والأوصياء على الأطفال

141. أذكر المحافظة التي تعيش بها *

Mark only one oval.

- الإسكندرية
- الإسماعيلية
- أسوان
- أسيوط
- الأقصر
- البحر الأحمر
- البحيرة
- بني سويف
- بورسعيد
- جنوب سيناء
- الجيزة
- الدقهلية
- دمياط
- سوهاج
- السويس
- الشرقية
- شمال سيناء
- الغربية
- الفيوم
- القاهرة
- القليوبية
- قنا

- كفر الشيخ
- مطروح
- المنوفية
- المنيا
- الوادي الجديد

142. الجنس/ النوع *

Mark only one oval.

- ذكر
- أنثى

143. العمر *

Mark only one oval.

- عام 18-29
- سنة 30-39
- سنة 40-49
- سنة 50-59
- أكثر من 60 سنة

144. صلة القرابة للطفل *

Mark only one oval.

- الأم
- الأب
- الجد/الجدة
- العم/ة أو الخال/ة
- غير ذلك

145. مستوى التعليم *

Mark only one oval.

- أمي لا يقرأ ولا يكتب
- يقرأ ويكتب
- ابتدائي
- اعدادي
- ثانوي
- جامعي
- دراسات عليا

146. محل العمل *

Mark only one oval.

- حكومي
- خاص
- لاأعمل

147. نوع العمل *

Mark only one oval.

- أعمل من خلال المنزل
- أعمل جزئيا (أذهب الي العمل 3 أو 4 مرات في الأسبوع)
- أذهب إلي العمل يوميا
- لاأعمل

148. هل تعمل بالمجال الصحي؟ *

Mark only one oval.

- نعم
- لا

149. هل لديك تأمين صحي؟ *

Mark only one oval.

- تأمين صحي حكومي
- تأمين صحي خاص
- بدون تـأمين صحي

150. الدخل الشهري *

Mark only one oval.

- لا يكفي ويستدين ولا يستطيع الوفاء بالدين
- لا يكفي ويستدين ويستطيع الوفاء بالدين
- يكفي
- يكفي ويدخر

151. هل يعيش أحد الوالدين أو الأقارب من كبار السن معك في نفس المنزل؟ *

Mark only one oval.

- نعم
- لا

152. ما هو عدد الأفراد الذين يعيشون بداخل منزلك؟ *

Mark only one oval.

- فردين
- 3 أفراد
- 4 أفراد
- 5 أفراد
- أكثر من 5 أفراد

153. هل تأكدت إصابتك بفيروس كورونا-19 من قبل؟ *

Mark only one oval.

- نعم
- لا
- غير متأكد

154. ما هو موقفك من لقاح كورونا-19؟ *

Mark only one oval.

- ( ) أرفض تلقي اللقاح (التطعيم)
- ( ) تلقيت الجرعة الأولي وبانتظار الجرعة الثانية
- ( ) تلقيت الجرعة الأولي ولن أتلقي الجرعة الثانية
- ( ) تلقيت الجرعة الأولي والثانية وفي انتظار الجرعة التنشيطية
- ( ) تلقيت الجرعة الأولي والثانية ولن أكمل الجرعة التنشيطية
- ( ) أتممت الجرعات (الأولي والثانية والتنشيطية)
- ( ) أوافق علي تلقي اللقاح وبانتظار تلقي الجرعات

155. ما هو سبب عدم استكمال جرعات اللقاح المضاد لفيروس كورونا-19؟ *

Check all that apply.

- [ ] لأن التطعيم غير متوفر
- [ ] لن استكمل الجرعات
- [ ] لأن ميعاد الجرعة الثانية من اللقاح لم يحن موعده بعد
- [ ] لا ينطبق

Other: [ ] ____________________

156. إن كنت تعاني من أمراض مزمنة, حدد تلك الأمراض *

Check all that apply.

- [ ] لا أعاني من أمراض مزمنة
- [ ] ضغط
- [ ] سكر
- [ ] قلب
- [ ] أمراض صدرية
- [ ] أمراض في الكلى والكبد
- [ ] أمراض روماتيزمية ومناعية
- [ ] أمراض نفسية وعصبية
- [ ] إعاقة حركية
- [ ] فقدان جزئي للسمع والبصر

Other: [ ] ______________________

البيانات الخاصة بالطفل الأول

157. ما هو عمر الطفل الأول؟ *

____

158. ما هو جنس/نوع الطفل الأول؟ *

Mark only one oval.

- ذكر
- انثى

159. هل سبق وأن تم تشخيص طفلك الأول بالإصابة بفيروس كورونا-19؟ *

Mark only one oval.

- نعم
- لا
- غير متأكد

160. هل يعاني طفلك الأول من أي أمراض مزمنة؟ (حدد تلك الأمراض) *

Check all that apply.

- [ ] لا يعاني أبني من أي أمراض مزمنة
- [ ] سكر
- [ ] ضغط
- [ ] قلب
- [ ] أمراض صدرية
- [ ] أمراض الكلى والكبد
- [ ] أمراض روماتيزمية ومناعية
- [ ] أمراض نفسية وعصبية
- [ ] إعاقات حركية
- [ ] فقدان جزئي للسمع أو البصر

161. هل تلقى طفلك الأول التطعيمات (اللقاحات) الإجبارية للأطفال من قبل وزارة الصحة المصرية؟ *

Mark only one oval.

- ( ) نعم
- ( ) لا
- ( ) غير متأكد

162. هل تلقى طفلك الأول تطعيم الانفلونزا الموسمية في خلال السنتين الماضيتين؟ *

Mark only one oval.

- نعم
- لا
- غير متأكد

163. ما هو موقفك تجاه تطعيم طفلك بتطعيم (لقاح) الكورونا-19؟ *

Mark only one oval.

- بالفعل تلقى طفلي تطعيم (لقاح) الكورونا-19
- أوافق على تطعيم طفلي ولكن تطعيم (لقاح) الكورونا-19 غيرمتوفر
- أرفض تطعيم طفلي بتطعيم (لقاح) كورونا-19

البيانات الخاصة بالطفل الثاني

164. ما هو عمر الطفل الثاني؟ *

165. ما هو جنس/نوع الطفل الثاني؟ *

Mark only one oval.

- ذكر
- انثى

166. هل سبق وأن تم تشخيص طفلك الثاني بالإصابة بفيروس كورونا-19؟ *

Mark only one oval.

- نعم
- لا
- غير متأكد

167. هل يعاني طفلك الثاني من أي أمراض مزمنة؟ (حدد تلك الأمراض) *

Check all that apply.

- [ ] لا يعاني أبني من أي أمراض مزمنة
- [ ] سكر
- [ ] ضغط
- [ ] قلب
- [ ] أمراض صدرية
- [ ] أمراض الكلى والكبد
- [ ] أمراض روماتيزمية ومناعية
- [ ] أمراض نفسية وعصبية
- [ ] إعاقات حركية
- [ ] فقدان جزئي للسمع أو البصر

168. هل تلقى طفلك الثاني التطعيمات (اللقاحات) الإجبارية للأطفال من قبل وزارة الصحة المصرية؟ *

Mark only one oval.

- ( ) نعم
- ( ) لا
- ( ) غير متأكد

169. هل تلقى طفلك الثاني تطعيم الانفلونزا الموسمية في خلال السنتين الماضيتين؟ *

Mark only one oval.

- نعم
- لا
- غير متأكد

170. ما هو موقفك تجاه تطعيم طفلك بتطعيم (لقاح) الكورونا-19؟ *

Mark only one oval.

- بالفعل تلقى طفلي تطعيم (لقاح) الكورونا-19
- أوافق على تطعيم طفلي ولكن تطعيم (لقاح) الكورونا-19 غيرمتوفر
- أرفض تطعيم طفلي بتطعيم (لقاح) كورونا-19

البيانات الخاصة بالطفل الثالث

171. ما هو عمر الطفل الثالث؟ *

172. ما هو جنس/نوع الطفل الثالث؟ *

Mark only one oval.

- ذكر
- انثى

173. هل سبق وأن تم تشخيص طفلك الثالث بالإصابة بفيروس كورونا-19؟ *

Mark only one oval.

- نعم
- لا
- غير متأكد

174. هل يعاني طفلك الثالث من أي أمراض مزمنة؟ (حدد تلك الأمراض) *

Check all that apply.

- [ ] لا يعاني أبني من أي أمراض مزمنة
- [ ] سكر
- [ ] ضغط
- [ ] قلب
- [ ] أمراض صدرية
- [ ] أمراض الكلى والكبد
- [ ] أمراض روماتيزمية ومناعية
- [ ] أمراض نفسية وعصبية
- [ ] إعاقات حركية
- [ ] فقدان جزئي للسمع أو البصر

175. هل تلقى طفلك الثالث التطعيمات (اللقاحات) الإجبارية للأطفال من قبل وزارة الصحة المصرية؟ *

Mark only one oval.

- ( ) نعم
- ( ) لا
- ( ) غير متأكد

176. هل تلقى طفلك الثالث تطعيم الانفلونزا الموسمية في خلال السنتين الماضيتين؟ *

Mark only one oval.

- نعم
- لا
- غير متأكد

177. ما هو موقفك تجاه تطعيم طفلك بتطعيم (لقاح) الكورونا-19؟ *

Mark only one oval.

- بالفعل تلقى طفلي تطعيم (لقاح) الكورونا-19
- أوافق على تطعيم طفلي ولكن تطعيم (لقاح) الكورونا-19 غيرمتوفر
- أرفض تطعيم طفلي بتطعيم (لقاح) كورونا-19

البيانات الخاصة بالطفل الرابع

178. ما هو عمر الطفل الرابع؟ *

179. ما هو جنس/نوع الطفل الرابع؟ *

Mark only one oval.

- ذكر
- انثى

180. هل سبق وأن تم تشخيص طفلك الرابع بالإصابة بفيروس كورونا-19؟ *

Mark only one oval.

- نعم
- لا
- غير متأكد

181. هل يعاني طفلك الرابع من أي أمراض مزمنة؟ (حدد تلك الأمراض) *

Check all that apply.

- [ ] لا يعاني أبني من أي أمراض مزمنة
- [ ] سكر
- [ ] ضغط
- [ ] قلب
- [ ] أمراض صدرية
- [ ] أمراض الكلى والكبد
- [ ] أمراض روماتيزمية ومناعية
- [ ] أمراض نفسية وعصبية
- [ ] إعاقات حركية
- [ ] فقدان جزئي للسمع أو البصر

182. هل تلقى طفلك الرابع التطعيمات (اللقاحات) الإجبارية للأطفال من قبل وزارة الصحة المصرية؟ *

Mark only one oval.

- ( ) نعم
- ( ) لا
- ( ) غير متأكد

183. هل تلقى طفلك الرابع تطعيم الانفلونزا الموسمية في خلال السنتين الماضيتين؟ *

Mark only one oval.

- نعم
- لا
- غير متأكد

184. ما هو موقفك تجاه تطعيم طفلك بتطعيم (لقاح) الكورونا-19؟ *

Mark only one oval.

- بالفعل تلقى طفلي تطعيم (لقاح) الكورونا-19
- أوافق على تطعيم طفلي ولكن تطعيم (لقاح) الكورونا-19 غيرمتوفر
- أرفض تطعيم طفلي بتطعيم (لقاح) كورونا-19

185. إذا أتيح لك الاختيار، هل ترغب في تطعيم أطفالك نوع محدد من اللقاحات المتوفرة ضد فيروس كورونا-19؟ *

Check all that apply.

- [ ] سينوفارم
- [ ] سينوفاك
- [ ] استرازينيكا
- [ ] سبوتنيك
- [ ] جونسون اند جونسون
- [ ] فايزر
- [ ] مودرنا
- [ ] أرفض تطعيم أطفالي
- [ ] لا أعلم

مقياس اتجاهات الآباء عن التطعيمات لأطفالهم

الرجاء اختيار إجابة واحدة

186. هل سبق لك في أي وقت أن أجلت تطعيم طفلك لأسباب غير المرض أو الحساسية؟ *

Mark only one oval.

- ( ) نعم
- ( ) لا
- ( ) لا أعلم

187. هل سبق لك أن قررت الامتناع عن تطعيم طفلك لأسباب غير المرض أو الحساسية؟ *

Mark only one oval.

- نعم
- لا
- لا أعلم

188. إلى أي مدى أنت متأكد أن الالتزام بجرعات التطعيم الموصي بها مفيدة بالنسبة لطفلك *

Mark only one oval.

	1	2	3	4	5	6	7	8	9	10	
غير متأكد علي الإطلاق											متأكد بشدة

189. يتلقى الأطفال عدد أكبر من جرعات التطعيم اللازمة لهم *

Mark only one oval.

- أوافق بشدة
- أوافق
- غير متأكد
- لا أوافق
- لا أوافق بشدة

190. أعتقد أن التطعيمات تقي من الأمراض الشديدة *

Mark only one oval.

- أوافق بشدة
- أوافق
- غير متأكد
- لا أوافق
- لا أوافق بشدة

191. من الأفضل لطفلي أن يكتسب مناعة عن طريق الإصابة بالمرض بدلا من أخذ التطعيم *

Mark only one oval.

- أوافق بشدة
- أوافق
- غير متأكد
- لا أوافق
- لا أوافق بشدة

192. من الأفضل أن يحصل الأطفال على عدد قليل من جرعات التطعيم في نفس الوقت *

Mark only one oval.

- أوافق بشدة
- أوافق
- غير متأكد
- لا أوافق
- لا أوافق بشدة

193. إلى أي مدى تشعر بالقلق من أن يسبب التطعيم عرض جانبي خطير لطفلك؟ *

Mark only one oval.

- أنا لست قلق أبدا تجاه ذلك
- أنا لست قلق تجاه ذلك
- غير متأكد
- أنا قلق قليلا تجاه ذلك
- أنا قلق كثيرا تجاه ذلك

194. إلى أي مدى تشعر بالقلق أن تكون احدى تطعيمات الاطفال غير امنة؟ *

Mark only one oval.

- ⬭ أنا لست قلق أبدا تجاه ذلك
- ⬭ أنا لست قلق تجاه ذلك
- ⬭ أنا قلق قليلا تجاه ذلك
- ⬭ أنا قلق كثيرا تجاه ذلك

195. إلى أي مدى تشعر بالقلق أن التطعيم لن يقي من المرض؟ *

Mark only one oval.

- ⬭ أنا لست قلق أبدا تجاه ذلك
- ⬭ أنا لست قلق تجاه ذلك
- ⬭ غير متأكد
- ⬭ أنا قلق قليلا تجاه ذلك
- ⬭ أنا قلق كثيرا تجاه ذلك

196. إذا رزقت بطفل في الوقت الحالي هل ترغب بأن يتلقى كل التطعيمات الموصى بها؟ *

Mark only one oval.

- نعم
- لا
- لا أعلم

197. عموما، الى أي درجة تعتبر نفسك متردد بشأن تطعيمات الأطفال؟ *

Mark only one oval.

- لست متردد أبدا
- لست متردد
- غير متأكد
- متردد
- متردد جدا

198. أثق بالمعلومات التي اتلقاها عن التطعيمات *

Mark only one oval.

- أوافق بشدة
- أوافق
- غير متأكد
- لا أوافق
- لا أوافق بشدة

199. أنا قادر على مناقشة مخاوفي الخاصة بالتطعيم مع طبيب طفلي *

Mark only one oval.

- أوافق بشدة
- أوافق
- غير متأكد
- لا أوافق
- لا أوافق بشدة

200. إجمالا، الى أي مدى تثق في طبيب طفلك؟ *

Mark only one oval.

	1	2	3	4	5	6	7	8	9	10	
لا أثق علي الإطلاق	⬭	⬭	⬭	⬭	⬭	⬭	⬭	⬭	⬭	⬭	أثق بشدة

نشكر تعاونكم وتقبلوا تحيات فريق البحث

This content is neither created nor endorsed by Google.

Google Forms
